# Development and external validation of a parsimonious lactate-to-diastolic blood pressure ratio model for 28-day mortality risk stratification in septic shock: a retrospective two-cohort study

**DOI:** 10.3389/fmed.2026.1827447

**Published:** 2026-06-15

**Authors:** Ziang Li, Wanglin Zhang, Kanlirong Wang, Tong Jin, Liqun Sun

**Affiliations:** The Second Affiliated Hospital of Nanjing Medical University, Nanjing, China

**Keywords:** diastolic blood pressure, external validation, lactate, prediction model, risk stratification, septic shock

## Abstract

**Background:**

Septic shock has high mortality and marked hemodynamic heterogeneity. Lactate is widely used for risk stratification, but it may underestimate risk when elevation is modest. We developed and externally validated a parsimonious model based on the lactate-to-invasive diastolic blood pressure ratio (LDR) to predict 28-day mortality in septic shock patients with invasive arterial monitoring.

**Methods:**

This retrospective two-cohort prediction-model study included 320 patients from an institutional ICU cohort and 962 from MIMIC-IV. T0 was defined as the start of continuous vasopressor infusion. LDR was calculated as lactate divided by invasive arterial DBP and reported as LDR × 100. The primary outcome was all-cause 28-day mortality after T0. Performance was assessed by discrimination, calibration, decision-curve analysis, lactate gray-zone performance, and four-quadrant phenotypes defined by lactate 4.0 mmol/L and DBP 50 mmHg. In MIMIC-IV, 1,619 of 2,581 clinically eligible patients were excluded because invasive DBP was unavailable; a noninvasive-only subgroup was analyzed to assess selection bias and generalizability.

**Results:**

Twenty-eight-day mortality was 29.7% in the development cohort and 26.0% in the validation cohort. LDR × 100 showed consistent discrimination, with AUCs of 0.726 (95% CI 0.657–0.786) and 0.714 (95% CI 0.674–0.753), respectively. Compared with lactate alone, LDR × 100 improved AUC in both cohorts, although the improvement was small in external validation (ΔAUC +0.029, 95% CI +0.006 to +0.051). In the lactate gray zone, external-validation discrimination was modest (AUC 0.598, 95% CI 0.533–0.664), supporting a complementary rather than stand-alone role. Patients with lactate <4.0 mmol/L and DBP <50 mmHg had higher mortality than those with lactate <4.0 mmol/L and DBP =50 mmHg, suggesting a possible high-risk subgroup. External validation showed calibration drift, indicating that absolute risk estimates require local recalibration. The noninvasive-only subgroup had higher mortality and lower LDR discrimination, supporting restriction to invasive-monitoring settings.

**Conclusion:**

LDR × 100 provides a simple bedside marker for rank-order risk stratification at vasopressor initiation in septic shock patients with invasive arterial monitoring. Its main potential value is complementary assessment in the lactate gray zone rather than replacement of established severity scores. Because of calibration drift, selection bias, and the observational design, LDR-based thresholds and treatment-guiding use require prospective validation before clinical adoption.

## Introduction

Septic shock is associated with high mortality and substantial pathophysiologic heterogeneity. Achievement of macrocirculatory targets does not necessarily indicate restoration of microcirculatory or cellular perfusion. Blood lactate is widely used for resuscitation monitoring and prognostication (1), and the Sepsis-3 definition of septic shock includes vasopressor use to maintain MAP ≥ 65 mmHg together with lactate > 2 mmol/L (2). However, lactate may increase because of adrenergic stress or impaired clearance and may not reliably capture real-time perfusion failure (3, 4).

The lactate gray zone (2.0− < 4.0 mmol/L) is particularly challenging because clinically meaningful risk may be underestimated when lactate is interpreted in isolation (5). Diastolic blood pressure (DBP) reflects vascular tone and vasoplegia, and both the diastolic shock index (DSI = HR/DBP) and low DBP have been associated with adverse outcomes in septic shock (6, 7). Thus, markedly low DBP may indicate severe vascular failure even when MAP appears acceptable.

We therefore investigated the lactate-to-invasive DBP ratio (LDR) as a simple bedside marker that integrates metabolic and vascular dysfunction in septic shock patients in whom invasive arterial pressure monitoring is already in place, and derived a parsimonious model based solely on LDR × 100. Our objectives were to develop the model in an institutional cohort, validate it externally in MIMIC-IV, compare its performance with lactate and established clinical scores, and assess its added value in the lactate gray zone. The model is therefore intended for risk stratification within this monitored subset rather than for septic shock patients in general.

## Materials and methods

### Study design and ethics

This retrospective, two-cohort prediction-model study followed TRIPOD (8), and the completed TRIPOD checklist is provided as Supplementary Material 2. The study was approved by the Ethics Committee of the Second Affiliated Hospital of Nanjing Medical University (No. 2026-KY-024-01), which waived informed consent for the development cohort. Use of MIMIC-IV complied with PhysioNet access requirements after CITI training and authorization (Certification No. 74434841) and was based on the published database documentation (9, 10).

### Data sources and study population

The development cohort included consecutive ICU patients treated at the Second Affiliated Hospital of Nanjing Medical University between June 2021 and December 2025. The external validation cohort consisted of first ICU stays recorded in MIMIC-IV v3.1 from 2008 to 2022.

Adults were clinically eligible if they met Sepsis-3 criteria for septic shock, had lactate > 2 mmol/L, required vasopressors after adequate fluid resuscitation, and started continuous vasopressor infusion within 0–180 min after lactate first exceeded 2 mmol/L. For the primary LDR analysis, we additionally required an invasive arterial DBP measurement recorded within 30 min before T0.

We excluded patients with shock predominantly due to non-septic causes, postoperative sepsis within 24 h of surgery, early comfort-focused care, or vasopressor exposure before T0. The complete SQL extraction code used for the MIMIC-IV validation cohort, including operational definitions of septic shock onset, vasopressor initiation (T0), the 0–180 min lactate window, the 30-min invasive DBP window, ICD-based exclusion criteria, mortality ascertainment, and SOFA/APACHE II component derivation, is provided in Supplementary material (LDR_extraction.sql, v3.9). De-identified development-cohort data and analysis code may be made available from the corresponding author upon reasonable request, subject to ethics approval and institutional data-sharing policies.

### Predictor and outcome definitions

T0 was defined as the start of continuous vasopressor infusion.

The primary predictor was LDR, defined as lactate (mmol/L) divided by invasive arterial DBP (mmHg), and reported as LDR × 100 for ease of interpretation. We used the lactate value closest to T0 within the preceding 0–180 min and the invasive arterial DBP value closest to T0 within the preceding 30 min. The asymmetric windows reflect the different temporal dynamics of the two measurements: arterial blood gases including lactate are typically sampled intermittently (every 1–3 h during early resuscitation), whereas invasive arterial pressure is recorded near-continuously, and lactate has a substantially longer plasma half-life than beat-to-beat DBP. Selecting the value closest to T0 in each case captures the patient’s physiologic state at vasopressor initiation while accommodating these sampling differences. A prespecified sensitivity analysis restricting lactate sampling to within 60 min before T0 yielded an essentially unchanged AUC in the validation cohort (Supplementary Tables 8–2), supporting the robustness of this window choice. Future prospective implementations should nonetheless aim for a 30–60-min pre-T0 lactate window when feasible, to minimize the residual timing heterogeneity inherent in the retrospective 0–180-min window.

The primary outcome was all-cause 28-day mortality after T0. Outcomes were obtained from hospital records and follow-up in the development cohort and from database death timestamps in the validation cohort.

### Model development, external validation, and performance assessment

We developed a logistic regression model with a single predictor (LDR × 100) and applied the original coefficients directly to the validation cohort. The parsimonious specification was prespecified for three reasons. First, lactate and invasive arterial DBP are immediately obtainable at T0 and require no retrospective aggregation, whereas integrated severity scores and comorbidity indices require additional data collection and are therefore less suitable for immediate bedside triage. Second, the development cohort included 95 events for a single predictor, which exceeds published sample-size requirements for prediction-model development and supports stable coefficient estimation with low risk of overfitting (11). Third, LDR × 100 is intended as a rapid risk-stratification trigger that complements—rather than replaces—comprehensive severity scores, and a parsimonious specification preserves this clinical role. As a sanity check, an exploratory multivariable extension with age, SOFA, NEE, and CCI is reported in Supplementary Table 12.

Discrimination was assessed using AUCs and paired DeLong tests (12).

Calibration was assessed using Brier scores and calibration plots (13–15). In external validation, we also estimated the calibration intercept and slope and explored logistic recalibration when drift was apparent (16).

Clinical utility was assessed by decision curve analysis (17).

Prespecified analyses focused on performance within the lactate gray zone (2.0 ≤ lactate < 4.0 mmol/L) and on a four-quadrant classification based on lactate 4.0 mmol/L and DBP 50 mmHg. NRI and IDI analyses are reported in Supplementary Material 1 (18).

### Sample size

We included all consecutive eligible patients during the study period (development *n* = 320; validation *n* = 962). Given the single-predictor model and the number of observed events, the development cohort met published sample-size guidance for prediction-model development (11), and the validation cohort (*n* = 962; 250 events) provided sufficient precision for estimating the calibration intercept, calibration slope, and discrimination in external validation.

#### Missing data and statistical software

The primary predictors (lactate, DBP, LDR × 100) and the primary outcome (28-day mortality), as well as age, SOFA, APACHE II, CCI, and NEE, were complete in both cohorts; heart rate at T0 was missing in 2.9% of validation-cohort patients. The primary analysis therefore used complete cases, which corresponded to the full eligible population for the predictors of interest. As a sensitivity analysis, multiple imputation by chained equations (m = 20 imputations) was applied to auxiliary variables used for descriptive and sensitivity analyses, including laboratory and physiologic variables with missingness such as platelets, creatinine, GCS, PaO_2_/FiO_2_ ratio, bilirubin, and temperature. Because lactate, invasive DBP, LDR × 100, and 28-day mortality had no missingness, imputation was not required for the primary LDR model; the univariate LDR model was re-estimated across imputed datasets to confirm equivalence with the complete-case analysis (19, 20). Analyses were performed using R and Python.

## Results

### Participants and baseline characteristics

**FIGURE 1:**
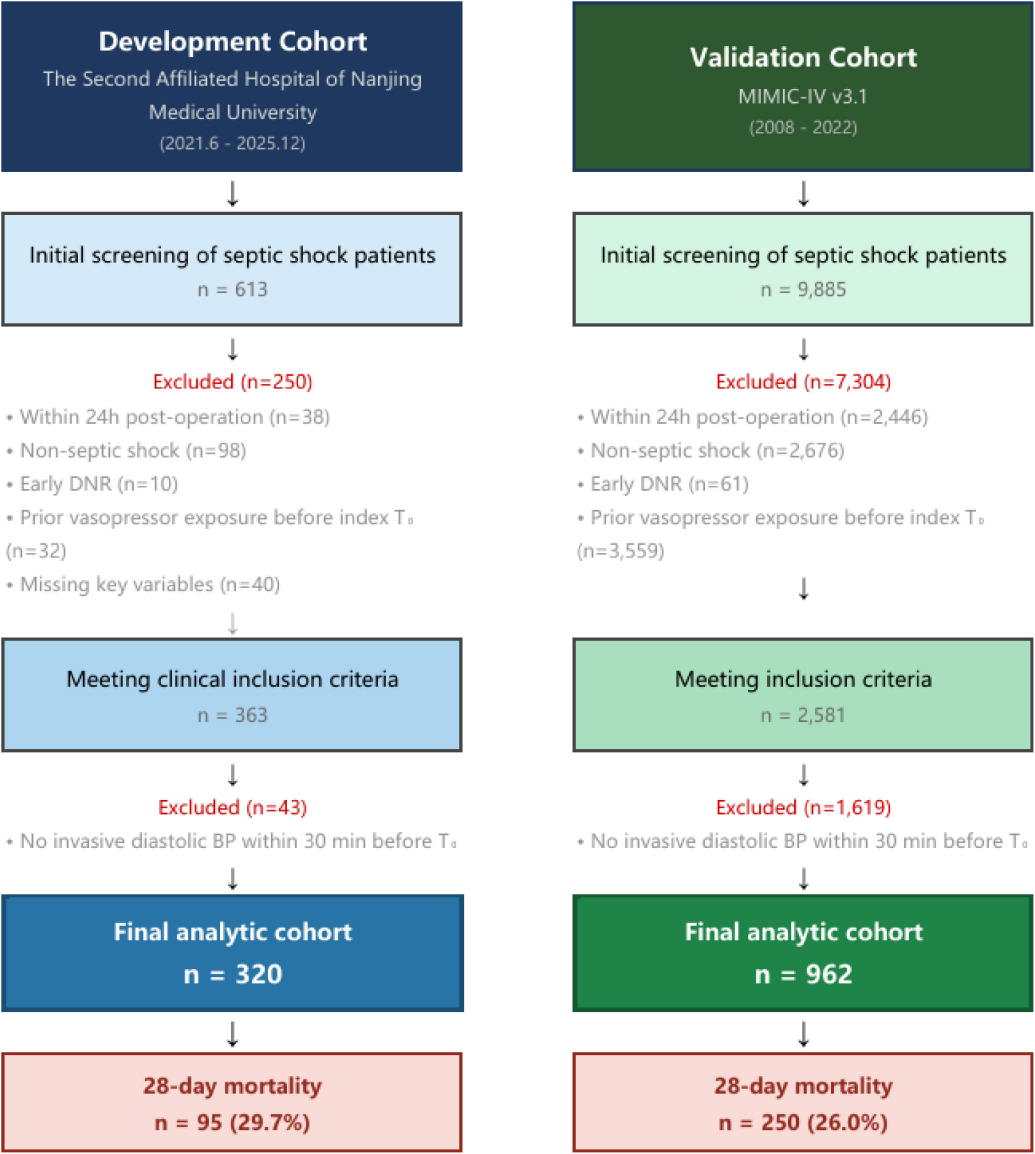
Flow diagram of patient selection in the development and validation cohorts. Development cohort: *n* = 320 (95 events, 28-day mortality 29.7%); external validation cohort (MIMIC-IV v3.1, 2008–2022): *n* = 962 (250 events, 28-day mortality 26.0%). The primary analysis required an invasive arterial DBP measurement within 30 min before T0; 1,619 of 2,581 clinically eligible MIMIC-IV patients lacking invasive monitoring within this window were excluded and analyzed separately as a noninvasive subgroup (Supplementary Material 1).

Of 613 screened patients, 320 were included in the development cohort; of 9,885 patients screened in MIMIC-IV, 962 were included in the validation cohort (Figure 1). Twenty-8-day mortality was 29.7% in the development cohort and 26.0% in the validation cohort. The distributions of lactate, DBP, and LDR × 100 were broadly similar across cohorts (Table 1).

**TABLE 1:** Baseline characteristics of the two cohorts (summary).

Characteristic	Development cohort (*n* = 320)	Validation cohort (*n* = 962)
Age (years)	67.9 ± 14.3	65.8 ± 15.5
Male, *n* (%)	201 (62.8%)	589 (61.2%)
SOFA score	8.9 ± 3.2	8.0 ± 3.0
APACHE II score	21.6 ± 7.6	21.0 ± 7.6
Lactate (mmol/L)	4.42 ± 2.99	4.37 ± 2.94
DBP (mmHg)	55.8 ± 13.0	55.3 ± 13.0
LDR × 100	8.44 ± 6.21	8.46 ± 7.20
CCI	4.7 ± 2.4	4.8 ± 2.7
28-day mortality, *n* (%)	95 (29.7%)	250 (26.0%)

Continuous variables are presented as mean ± standard deviation. There were no missing data for the main analysis variables (lactate, DBP, LDR × 100) or the primary outcome; missingness for other variables is summarized in Supplementary Tables 5-1, 5-2.

In the MIMIC-IV cohort, 1,619 of 2,581 clinically eligible patients were excluded from the primary validation analysis because invasive DBP was unavailable within 30 min before T0. Among these excluded patients, 844 had noninvasive cuff DBP recorded at T0 and were analyzed separately as a noninvasive-only monitoring subgroup to assess selection bias and generalizability. Compared with the invasive-monitoring cohort, this group had greater DBP variability (31.9% vs. 21.9%), lower LDR discrimination (AUC 0.633 vs. 0.714; ΔAUC = 0.081), and higher mortality (48.6% vs. 26.0%). These findings support the use of LDR primarily in settings with invasive arterial monitoring (Supplementary Tables 1-1, 1-2). The exclusion of patients without invasive monitoring introduces selection bias: this group had higher mortality (48.6% vs. 26.0%), and the model’s performance in their broader population is therefore likely overstated. We address this in two ways. First, the model is intended for use in patients in whom invasive arterial monitoring is already indicated for hemodynamic management, not as a population-wide screening tool. Second, sensitivity analyses applying LDR to the noninvasive subgroup confirmed reduced discrimination (AUC 0.633), which we report transparently rather than as the primary result. Generalizability to settings without invasive monitoring requires dedicated prospective evaluation.

### Model discrimination and external validation

**FIGURE 2:**
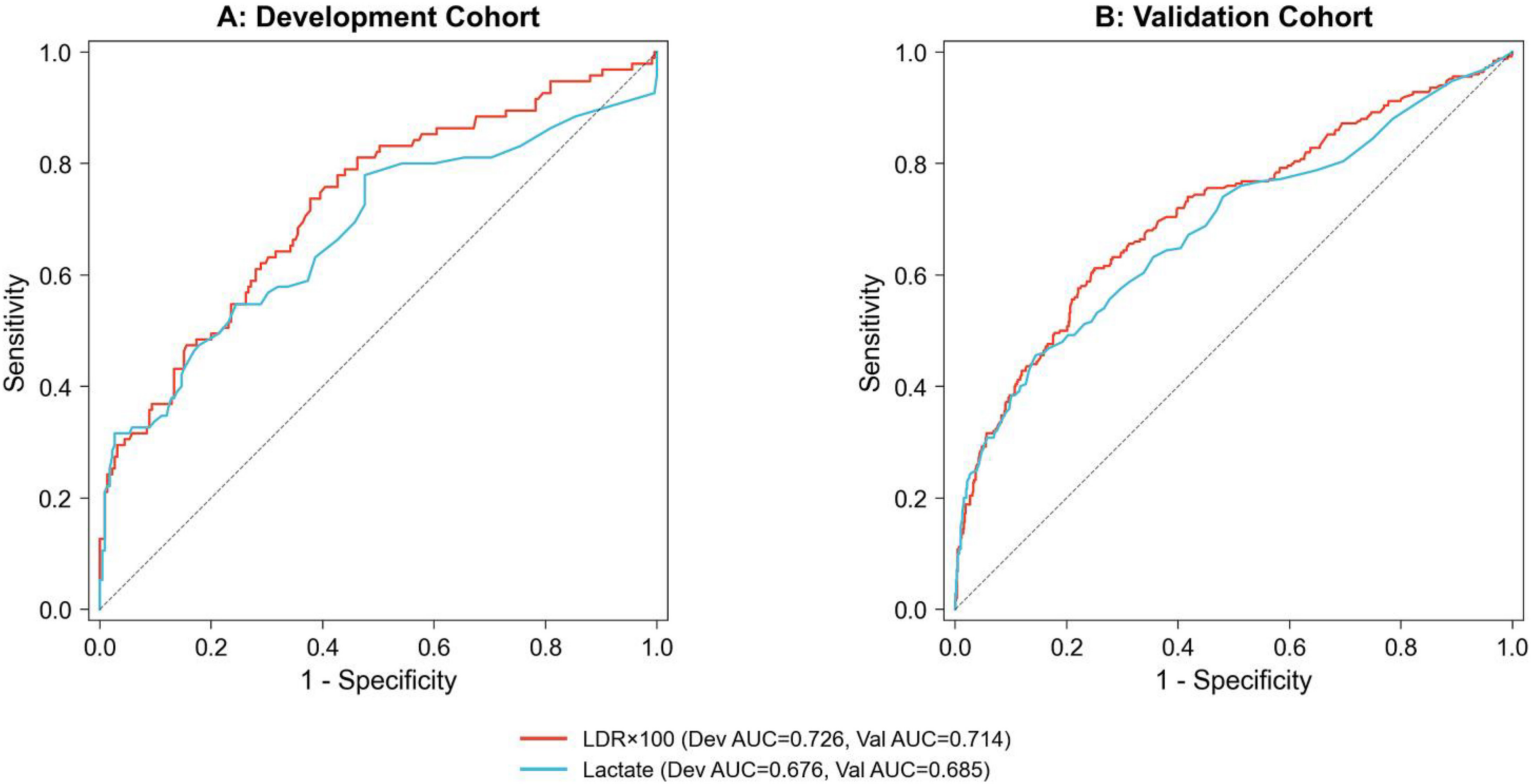
ROC curves comparing LDR × 100 with lactate alone for prediction of 28-day mortality in the development [**(A)**; *n* = 320] and validation [**(B)**; *n* = 962] cohorts. Development cohort: AUC LDR × 100 = 0.726 (95% CI 0.663–0.787), AUC lactate = 0.676 (95% CI 0.606–0.749), ΔAUC = +0.050 (95% CI +0.014 to +0.085, *P* = 0.006, DeLong). Validation cohort: AUC LDR × 100 = 0.714 (95% CI 0.673–0.754), AUC lactate = 0.685 (95% CI 0.642–0.726), ΔAUC = +0.029 (95% CI +0.006 to +0.051, *P* = 0.012). All 95% CIs were obtained by 2,000-iteration nonparametric bootstrap.

LDR × 100 showed consistent discrimination for 28-day mortality, with an AUC of 0.726 (95% CI 0.657–0.786) in the development cohort and 0.714 (95% CI 0.674–0.753) in the validation cohort (Table 2 and Figure 2).

**TABLE 2:** Key model performance metrics (primary analysis).

Metric	Development cohort	Validation cohort
AUC: LDR × 100 (Model 1)	0.726 (0.663–0.787)	0.714 (0.674–0.753)
AUC: lactate	0.676 (0.604–0.747)	0.685 (0.644–0.727)
ΔAUC (LDR × 100–lactate)	+0.050 (+0.014 to +0.085, *P* = 0.006)	+0.029 (+0.006 to +0.051, *P* = 0.012)
AUC: SOFA	0.734 (0.675–0.792)	0.687 (0.648–0.725)
AUC: APACHE II	0.717 (0.656–0.776)	0.713 (0.677–0.749)
Brier (Model 1)	0.173 (0.149–0.197)	0.165 (0.152–0.179)
Calibration intercept (Model 1)	–	−0.335 (−0.600 to −0.045)
Calibration slope (Model 1)	–	0.830 (0.588–1.110)
Gray-zone AUC: LDR × 100	0.678 (0.567–0.780)	0.598 (0.533–0.664)
Gray-zone AUC: lactate	0.540 (0.432–0.651)	0.530 (0.463–0.594)

Values in brackets are 95% confidence intervals; for AUC values these were obtained by paired DeLong methods, and for ΔAUC, Brier score, calibration intercept/slope, and gray-zone AUCs by 2,000-iteration nonparametric bootstrap. The gray zone was defined as 2.0 ≤ lactate < 4.0 mmol/L. Paired DeLong comparisons with SOFA and APACHE II are provided in Supplementary Table 4. Diagnostic performance at the LDR × 100 threshold of 6.45 is provided in Supplementary Table 6. The lactate-T0 time-window sensitivity analysis and heart rate-limited subgroup analysis are shown in Supplementary Tables 8–2, 9–1, respectively. Additional supplementary analyses, including NRI/IDI, are provided in Supplementary Material 1.

Compared with lactate alone, LDR × 100 improved AUC in both cohorts (development ΔAUC = +0.050, 95% CI +0.014 to +0.085, *P* = 0.006; validation ΔAUC = +0.029, 95% CI +0.006 to +0.051, *P* = 0.012) and performed similarly to SOFA and APACHE II (Table 2 and Supplementary Table 4). Although these improvements were statistically significant, the absolute increment—particularly the +0.029 observed in the external validation cohort—is small, and its clinical relevance should not be inferred from statistical significance alone; the practical value of LDR × 100 rests primarily on its immediate bedside availability rather than on a large gain in discrimination over lactate.

### Model specification and calibration

The final model was logit(p) = −2.365 + 0.172 × (LDR × 100).

**FIGURE 3:**
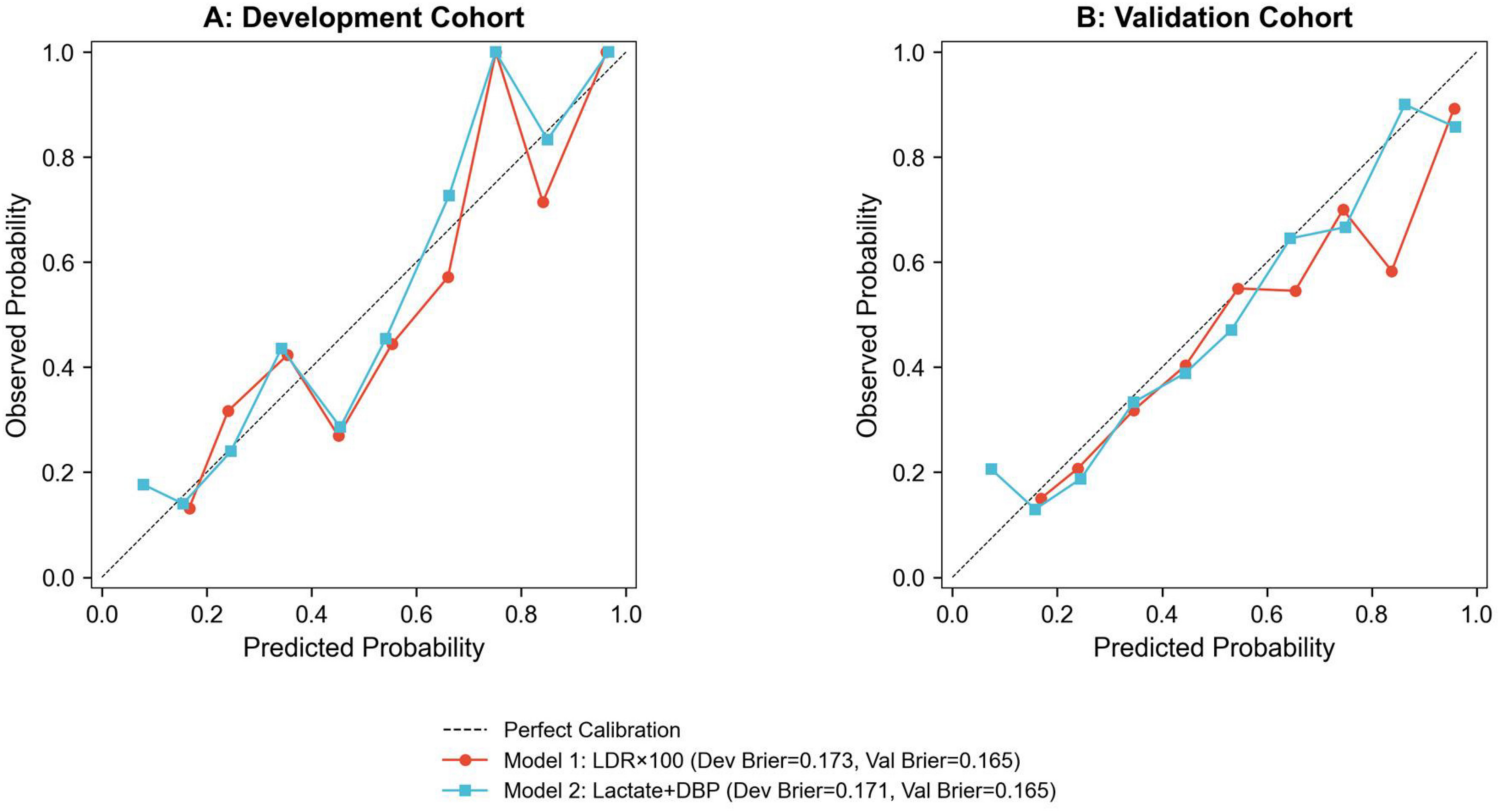
Calibration curves for the LDR × 100 model in **(A)** the development cohort (*n* = 320) and **(B)** the validation cohort (*n* = 962). Brier score: development 0.173 (95% CI 0.149–0.197); validation 0.165 (95% CI 0.152–0.179). Calibration intercept in validation = –0.335 (95% CI –0.600 to –0.045), calibration slope = 0.830 (95% CI 0.588–1.110), indicating calibration drift on external validation: the original model systematically miscalibrates absolute risk in new populations and should be used for rank-order risk stratification only, with local recalibration required before LDR × 100 is used to estimate absolute 28-day mortality risk (see “Results,” and Supplementary Material 1). All 95% CIs were obtained by 2,000-iteration nonparametric bootstrap.

The Brier score was 0.173 (95% CI 0.149–0.197) in the development cohort and 0.165 (95% CI 0.152–0.179) in the validation cohort. External validation showed calibration drift (intercept −0.335, 95% CI −0.600 to −0.045; slope 0.830, 95% CI 0.588–1.110), indicating that the original model systematically miscalibrates absolute risk in the validation setting. Absolute risk predictions from the development-cohort coefficients should therefore not be used directly in new populations: the original model should be used for rank-order risk stratification only, and local recalibration is required before LDR × 100 is used to estimate absolute 28-day mortality risk for clinical decision-making. When absolute risk estimates are required, the recalibrated linear predictor may be used (Figure 3 and Supplementary Material 1).

For settings in which absolute risk estimation is needed, the linear predictor can be _*recal*_ibrated as LP_recal = −0.335 + 0.830 × LP, and the recalibrated probability is p_recal = 1/[1 + exp(−LP_recal)].

### Incremental value in the lactate gray zone and the hidden high-risk phenotype

Within the lactate gray zone, LDR × 100 showed a numerically larger advantage over lactate alone than in the overall cohort (development AUC 0.678 vs. 0.540; validation 0.598 vs. 0.530; Table 2). Although the relative improvement was greater than in the full cohort, absolute discrimination in the gray zone was modest, particularly in the validation cohort (AUC 0.598), and the gray-zone results should be interpreted as supporting a complementary risk-stratification role rather than establishing LDR × 100 as a stand-alone discriminator in this subgroup.

In the four-quadrant analysis, the hidden high-risk phenotype (Q2: lactate < 4.0 mmol/L and DBP < 50 mmHg) had substantially higher mortality than the reference group Q4 (development 36.2% vs. 14.3%, OR 3.41; validation 25.5% vs. 13.3%, OR 2.24; Tables 3A,B). After adjustment for age, SOFA, NEE, and CCI, the Q2 phenotype remained independently associated with 28-day mortality (development adjusted OR 2.80, 95% CI 1.28–6.11, *P* = 0.010; validation adjusted OR 1.89, 95% CI 1.19–3.01, *P* = 0.007), supporting that the signal is not solely explained by overall illness severity.

**TABLE 3A:** Four-quadrant phenotypes and 28-day mortality risk—development cohort.

Quadrant	*n*	Mortality	OR (95% CI)
Q4: lactate < 4 and DBP ≥ 50	140	14.3%	Ref
Q2: lactate < 4 and DBP < 50	58	36.2%	3.41 (1.67–6.96)
Q3: lactate ≥ 4 and DBP ≥ 50	80	37.5%	3.60 (1.87–6.93)
Q1: lactate ≥ 4 and DBP < 50	42	57.1%	8.00 (3.69–17.33)

**TABLE 3B:** Four-quadrant phenotypes and 28-day mortality risk—external validation cohort.

Quadrant	*n*	Mortality	OR (95% CI)
Q4: lactate < 4 and DBP ≥ 50	392	13.3%	Ref
Q2: lactate < 4 and DBP < 50	200	25.5%	2.24 (1.45–3.45)
Q3: lactate ≥ 4 and DBP ≥ 50	247	34.4%	3.43 (2.32–5.08)
Q1: lactate ≥ 4 and DBP < 50	123	50.4%	6.65 (4.20–10.51)

Note: Quadrants were defined by lactate × DBP. Cutoffs were lactate 4.0 mmol/L and DBP 50 mmHg; Q4 served as the reference category for OR estimation.

**FIGURE 4:**
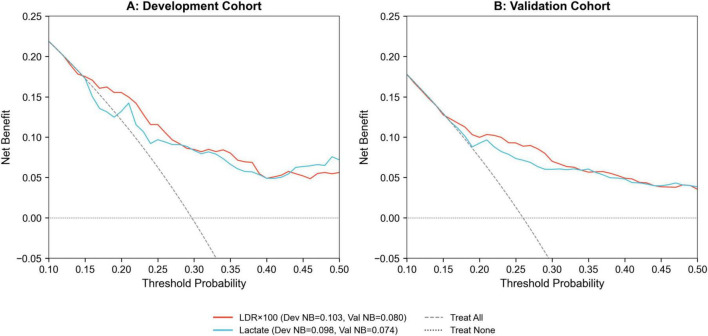
Decision curve analysis comparing the net benefit of the LDR × 100 model (Model 1) with that of a lactate-only strategy in **(A)** the development cohort and **(B)** the validation cohort. Net-benefit estimates are presented without confidence intervals because decision-curve net-benefit values are not routinely reported with uncertainty intervals; differences should therefore be interpreted as exploratory descriptions of potential utility under stated threshold preferences, not as evidence of clinical benefit from LDR-guided management, which would require a prospective interventional study. The lactate gray zone for incremental-value analyses (Table 2 and Supplementary Tables 6, 7) was defined as 2.0 ≤ lactate < 4.0 mmol/L.

Decision curve analysis showed numerically greater net benefit for Model 1 than for a lactate-only strategy across the threshold-probability ranges examined (approximately 12%–47% in the development cohort and 12%–40% in the validation cohort; Figure 4). Because decision-curve net-benefit estimates do not carry confidence intervals as routinely reported, these differences should be interpreted as exploratory; they describe potential utility under stated threshold preferences rather than demonstrate clinical benefit from LDR-guided management, which would require a prospective interventional study.

Results were robust in sensitivity analyses: restricting lactate sampling to within 60 min before T0 yielded an AUC of 0.721 in the validation cohort, compared with 0.714 in the full cohort (Supplementary Table 8–2).

Using the development-cohort Youden-derived threshold of LDR × 100 = 6.45, sensitivity and specificity in the validation cohort were 72.0% and 59.4% in the full cohort, and 35.0% and 82.2% in the gray-zone subgroup (Supplementary Table 6). Because this threshold was selected by maximizing Youden’s J in the development cohort, the reported operating characteristics are optimistic, and the cutoff should be regarded as exploratory pending prospective validation rather than as a definitive clinical decision rule.

## Discussion

In this two-cohort study, a model based solely on LDR × 100 provided consistent discrimination for 28-day mortality at vasopressor initiation in septic shock and showed good transportability from the derivation cohort to MIMIC-IV.

LDR × 100 outperformed lactate alone in both cohorts, with the greatest advantage in the lactate gray zone. These findings suggest that incorporating vascular-tone information refines risk assessment when lactate elevation is modest.

The four-quadrant analysis translated this finding into a clinically intuitive phenotype: patients with lactate < 4 mmol/L but DBP < 50 mmHg may represent a hidden high-risk subgroup. Such patients may warrant closer monitoring and reassessment, although our observational data do not demonstrate that phenotype-guided treatment improves outcomes.

LDR is not intended to replace comprehensive severity scores such as SOFA or APACHE II. SOFA and APACHE II showed AUCs comparable to LDR × 100 (Table 2), but require aggregation of multiple physiologic and laboratory variables and are generally not available at the moment of vasopressor initiation; the principal practical advantage of LDR × 100 is therefore its immediate availability at the bedside from two routinely monitored variables, rather than superior discrimination *per se*. A practical, development-cohort-derived threshold is LDR × 100 ≥ 6.45 (equivalently, DBP ≤ lactate × 15.5 mmHg), identifying a higher-risk stratum within the cohort whose absolute predicted risk varies with the recalibrated linear predictor (Supplementary Tables 6, 11). At this threshold, clinicians might prioritize (i) earlier reassessment of the resuscitation strategy, including consideration of additional vasoactive support targeting vascular tone—as LDR × 100 ≥ 6.45 signals disproportionate vasodilation relative to metabolic stress (DBP already low at a lactate level that is not yet extreme), a profile distinct from the tissue-hypoperfusion signal of isolated lactate elevation; (ii) more frequent perfusion reassessment; and (iii) earlier escalation discussions with the responsible team. We emphasize that this is hypothesis-generating: our observational data do not establish that decisions triggered by LDR × 100 ≥ 6.45 improve outcomes, and the threshold was derived using Youden’s index in the development cohort and requires prospective validation before being adopted as a definitive clinical cutoff. Detailed diagnostic metrics and a bedside conversion table are provided in Supplementary Table 6, 7; local recalibration is preferable when absolute risk estimates are needed.

LDR showed greater discrimination than DSI in patients with limited heart rate response (HR < 100 beats/min), suggesting that it may be more robust when the heart-rate response is blunted, such as during beta-blocker exposure (Supplementary Table 9-1).

This study has several limitations. First, the retrospective design and reliance on routinely collected electronic data carry inherent risks of residual confounding and unmeasured-variable bias; we cannot exclude that the observed associations are partly driven by factors not captured in either cohort. Second, the primary analysis required an invasive arterial DBP measurement within 30 min before T0, and 1,619 of 2,581 clinically eligible MIMIC-IV patients were therefore excluded. This invasive-monitoring requirement introduces selection bias: excluded patients had higher 28-day mortality (48.6% vs. 26.0%) and LDR × 100 showed lower discrimination in the noninvasive subgroup (AUC 0.633 vs. 0.714). The higher mortality in the noninvasive subgroup does not appear to reflect more acute physiologic severity but a different patient mix—noninvasively monitored patients were substantially more likely to carry dementia, to have been admitted from a long-term-care facility, to have end-stage renal disease, and to be transitioned to comfort-focused care during the admission (Supplementary Table 1–3)—consistent with a population in whom death is driven more by chronic illness trajectory and goals-of-care decisions than by the acute hemodynamic-metabolic decompensation that LDR is designed to capture. The model’s reported performance therefore applies specifically to ICUs with invasive arterial monitoring already in place, and generalizability to settings without invasive monitoring is not established. Third, lactate and DBP measurements were drawn from asymmetric pre-T0 windows (0–180 min and 0–30 min, respectively), introducing timing heterogeneity that could not be fully controlled despite the prespecified 60-min sensitivity analysis. Fourth, the development cohort spans 2021–2025 and the MIMIC-IV validation cohort spans 2008–2022; secular changes in sepsis recognition, resuscitation practice (including fluid and vasopressor strategy), and supportive care across this treatment era may contribute to the observed calibration drift and may also affect generalizability. Fifth, the LDR × 100 threshold of 6.45 was derived using Youden’s index in the development cohort and has not undergone prospective validation; reported sensitivity and specificity at this cutoff are therefore optimistic and should be interpreted as preliminary. Finally, this study is observational and was not designed to evaluate clinical interventions: we provide no evidence that LDR-guided management improves patient outcomes, and the proposed clinical actions at LDR × 100 ≥ 6.45 should be regarded as hypothesis-generating until tested in a prospective interventional study.

Prospective multicenter studies are needed to validate the threshold, evaluate recalibration strategies, and determine whether targeted interventions for the Q2 phenotype improve prognosis.

## Conclusion

LDR × 100, derived from lactate and invasive DBP, provided rapid risk stratification for 28-day mortality at vasopressor initiation in septic shock. Its principal observation of potential clinical interest was a possible hidden high-risk subgroup within the lactate gray zone that might be overlooked when lactate is interpreted in isolation; this finding is hypothesis-generating and requires prospective confirmation. The model appears best suited to ICUs with invasive arterial monitoring and may require local recalibration when used for absolute risk prediction.

## Data Availability

The original contributions presented in this study are included in this article/Supplementary material, further inquiries can be directed to the corresponding author.

## Supplementary material

Supplementary Material 1 Additional methods, tables, and figures (including data extraction and variable definitions, additional head-to-head comparisons, threshold diagnostic performance, and sensitivity analyses).

Supplementary Material 2 TRIPOD checklist (completed).
