# Supplementary material for: Development and external validation of a parsimonious lactate-to-diastolic blood pressure ratio model for 28-day mortality risk stratification in septic shock: a retrospective two-cohort study
Source: Front Med (Lausanne). 2026 Jun 15;13:1827447. doi: 10.3389/fmed.2026.1827447 (PMC13311116; doi:10.3389/fmed.2026.1827447)
Supplement: Supplementary file 1 [file Table_3.docx]

-- ============================================================================

-- LDR_extraction.sql v3.9 (invasive arterial blood pressure version)

-- ============================================================================

-- Septic shock cohort extraction with SOFA and APACHE II scoring

-- for MIMIC-IV v3.1 external validation

--

-- v3.9 fixes:

-- [Fix 1] admission_status: removed adm.admission_type from GROUP BY,

-- replaced with MAX aggregate

-- [Fix 2] Final JOIN: charlson table deduplicated before JOIN

-- [Fix 3] All CTEs annotated with uniqueness guarantees

-- [New] sbp_at_t0, hr_at_t0 (scalar subqueries, guaranteed single-row)

-- ============================================================================

-- Invasive arterial BP version:

-- DBP: itemid 220051 (invasive diastolic blood pressure)

-- SBP: itemid 220050 (invasive systolic blood pressure)

-- Time window unchanged: 30 minutes before T0

-- ============================================================================

-- ============================================================================

-- Step 0: Create indexes on base tables (if not already present)

-- ============================================================================

CREATE INDEX IF NOT EXISTS idx_labevents_subject_item_time

ON mimiciv_hosp.labevents(subject_id, itemid, charttime);

CREATE INDEX IF NOT EXISTS idx_chartevents_stay_item_time

ON mimiciv_icu.chartevents(stay_id, itemid, charttime);

CREATE INDEX IF NOT EXISTS idx_inputevents_stay_item_start

ON mimiciv_icu.inputevents(stay_id, itemid, starttime);

CREATE INDEX IF NOT EXISTS idx_diagnoses_hadm_version

ON mimiciv_hosp.diagnoses_icd(hadm_id, icd_version, icd_code);

CREATE INDEX IF NOT EXISTS idx_chemistry_hadm_time

ON mimiciv_derived.chemistry(hadm_id, charttime);

CREATE INDEX IF NOT EXISTS idx_enzyme_hadm_time

ON mimiciv_derived.enzyme(hadm_id, charttime);

CREATE INDEX IF NOT EXISTS idx_bg_hadm_time_specimen

ON mimiciv_derived.bg(hadm_id, charttime, specimen);

CREATE INDEX IF NOT EXISTS idx_vitalsign_stay_time

ON mimiciv_derived.vitalsign(stay_id, charttime);

CREATE INDEX IF NOT EXISTS idx_cbc_hadm_time

ON mimiciv_derived.complete_blood_count(hadm_id, charttime);

CREATE INDEX IF NOT EXISTS idx_gcs_stay_time

ON mimiciv_derived.gcs(stay_id, charttime);

-- ============================================================================

-- Main query

-- ============================================================================

DROP TABLE IF EXISTS mimiciv_derived.septic_shock_cohort_with_scores;

CREATE TABLE mimiciv_derived.septic_shock_cohort_with_scores AS

-- ============================================================================

-- Step 1: Base cohort from the sepsis3 derived table

-- [Uniqueness] sepsis3.stay_id is unique; icustays.stay_id is a primary key

-- ============================================================================

WITH sepsis3_base AS (

SELECT

s3.subject_id,

s3.stay_id,

s3.sofa_time AS sepsis_onset_time,

ie.intime AS icu_intime,

ie.outtime AS icu_outtime,

ie.hadm_id

FROM mimiciv_derived.sepsis3 s3

INNER JOIN mimiciv_icu.icustays ie ON s3.stay_id = ie.stay_id

WHERE s3.sepsis3 = TRUE

),

-- ============================================================================

-- Step 2: Lactate > 2 mmol/L records

-- [Expected multi-row] A patient may have multiple lactate measurements

-- ============================================================================

lactate_elevated AS (

SELECT

sb.subject_id,

sb.hadm_id,

sb.stay_id,

sb.sepsis_onset_time,

sb.icu_intime,

le.charttime AS lactate_time,

le.valuenum AS lactate

FROM sepsis3_base sb

INNER JOIN mimiciv_hosp.labevents le

ON sb.subject_id = le.subject_id

AND (le.hadm_id = sb.hadm_id OR le.hadm_id IS NULL)

AND le.charttime >= sb.icu_intime - INTERVAL '2 hours'

AND le.charttime <= sb.icu_outtime

WHERE le.itemid IN (50813, 52442)

AND le.valuenum > 2

AND le.valuenum < 50

AND le.valuenum IS NOT NULL

),

-- ============================================================================

-- Step 3: Vasopressor use

-- [Expected multi-row] A patient may receive multiple vasopressors

-- ============================================================================

vasopressor_use AS (

SELECT

inp.stay_id,

inp.starttime AS vaso_starttime,

inp.endtime AS vaso_endtime,

inp.itemid,

inp.rate

FROM mimiciv_icu.inputevents inp

WHERE EXISTS (SELECT 1 FROM sepsis3_base sb WHERE sb.stay_id = inp.stay_id)

AND inp.itemid IN (221906, 221289, 221662, 221749, 222315)

AND inp.rate > 0

AND (inp.statusdescription IS NULL

OR inp.statusdescription NOT IN ('Rewritten', 'Stopped', 'Paused', 'Flushed'))

),

-- ============================================================================

-- Step 4: Define T0 (vasopressor initiation time)

-- [Uniqueness] ROW_NUMBER ensures one row per (subject_id, stay_id)

-- ============================================================================

t0_candidates_raw AS (

SELECT

lac.subject_id,

lac.hadm_id,

lac.stay_id,

lac.sepsis_onset_time,

lac.lactate_time AS trigger_lactate_time,

lac.lactate AS trigger_lactate,

vaso.vaso_starttime AS t0_time,

vaso.itemid AS first_vaso_itemid,

(EXTRACT(EPOCH FROM (lac.lactate_time - vaso.vaso_starttime)) / 60.0)::NUMERIC(10,2) AS lactate_to_t0_minutes,

ROW_NUMBER() OVER (

PARTITION BY lac.subject_id, lac.stay_id, lac.lactate_time

ORDER BY vaso.vaso_starttime ASC

) AS rn_per_lactate

FROM lactate_elevated lac

INNER JOIN vasopressor_use vaso

ON lac.stay_id = vaso.stay_id

AND vaso.vaso_starttime >= lac.lactate_time - INTERVAL '0 minutes'

AND vaso.vaso_starttime <= lac.lactate_time + INTERVAL '180 minutes'

),

t0_candidates_filtered AS (

SELECT *

FROM t0_candidates_raw

WHERE rn_per_lactate = 1

),

t0_candidates AS (

-- [Uniqueness] PARTITION BY subject_id, stay_id + rn_final = 1

SELECT

subject_id, hadm_id, stay_id, sepsis_onset_time,

trigger_lactate_time, trigger_lactate, t0_time, first_vaso_itemid

FROM (

SELECT *,

ROW_NUMBER() OVER (

PARTITION BY subject_id, stay_id

ORDER BY

t0_time ASC,

CASE WHEN trigger_lactate_time <= t0_time THEN 0 ELSE 1 END,

ABS(lactate_to_t0_minutes),

trigger_lactate DESC,

first_vaso_itemid ASC

) AS rn_final

FROM t0_candidates_filtered

) best_t0

WHERE rn_final = 1

),

-- ============================================================================

-- Step 5: Exclusion criteria (ICD-based and behavioral)

-- ============================================================================

-- Scan all ICD diagnosis codes for exclusion flags

combined_diag_scan AS (

-- [Uniqueness] GROUP BY hadm_id

SELECT

d.hadm_id,

-- Non-septic shock and other exclusion diagnoses

MAX(CASE WHEN

(d.icd_version = 9 AND (

-- 1. Acute myocardial infarction (AMI)

d.icd_code LIKE '410%'

-- 2. Pericardial effusion

OR d.icd_code LIKE '4232%'

-- 3. Hypovolemic shock

OR d.icd_code LIKE '78559%'

-- 4. Pulmonary embolism

OR d.icd_code LIKE '4151%'

-- 5. Burns: not excluded (cohort had only minor burns, likely infection source)

-- 6. Acute spinal cord injury

OR d.icd_code LIKE '952%' OR d.icd_code LIKE '953%' OR d.icd_code LIKE '806%'

-- 7. Aortic dissection (obstructive shock)

OR d.icd_code LIKE '441.0%' OR d.icd_code LIKE '4410%'

-- 8. Esophageal-variceal bleeding (hypovolemic shock)

OR d.icd_code LIKE '456.0%' OR d.icd_code LIKE '4560%'

OR d.icd_code LIKE '456.2%' OR d.icd_code LIKE '4562%'

-- 9. Postpartum hemorrhage (hypovolemic shock)

OR d.icd_code LIKE '666%'

-- 10. Adrenal crisis (distributive shock, endocrine)

OR d.icd_code LIKE '255.41%' OR d.icd_code LIKE '25541%'

)) OR

(d.icd_version = 10 AND (

-- 1. Acute myocardial infarction (AMI)

d.icd_code LIKE 'I21%' OR d.icd_code LIKE 'I22%'

-- 2. Pericardial effusion

OR d.icd_code LIKE 'I313%'

-- 3. Hypovolemic shock

OR d.icd_code LIKE 'R571%'

-- 4. Pulmonary embolism

OR d.icd_code LIKE 'I26%'

-- 5. Burns: not excluded (see above)

-- 6. Heatstroke

OR d.icd_code LIKE 'T670%'

-- 7. Acute spinal cord injury

OR d.icd_code LIKE 'S14%' OR d.icd_code LIKE 'S24%' OR d.icd_code LIKE 'S34%'

-- 8. Aortic dissection (obstructive shock)

OR d.icd_code LIKE 'I710%' OR d.icd_code LIKE 'I71.0%'

-- 9. Esophageal-variceal bleeding (hypovolemic shock)

OR d.icd_code LIKE 'I850%' OR d.icd_code LIKE 'I85.0%'

OR d.icd_code LIKE 'I864%' OR d.icd_code LIKE 'I86.4%'

-- 10. Postpartum hemorrhage (hypovolemic shock)

OR d.icd_code LIKE 'O72%'

-- 11. Adrenal crisis (distributive shock, endocrine)

OR d.icd_code LIKE 'E272%' OR d.icd_code LIKE 'E27.2%'

))

THEN 1 ELSE 0 END) AS is_excluded_non_dnr,

-- Conditions potentially affecting lactate metabolism

-- NOTE: This flag is scanned for descriptive purposes but is NOT applied

-- as a final exclusion criterion (see t0_filtered below). Excluding these

-- conditions (epilepsy, alcohol-related liver disease, cirrhosis, DKA)

-- would remove a clinically relevant subset of septic shock patients

-- in whom LDR is still intended to be used.

MAX(CASE WHEN

(d.icd_version = 9 AND (

-- Epilepsy / seizures (muscle activity producing lactate)

d.icd_code LIKE '345%' OR d.icd_code LIKE '7803%'

-- Alcohol-related conditions (impaired gluconeogenesis, lactate clearance)

OR d.icd_code LIKE '303%' OR d.icd_code LIKE '305.0%'

OR d.icd_code LIKE '571.0%' OR d.icd_code LIKE '571.1%'

OR d.icd_code LIKE '571.2%' OR d.icd_code LIKE '571.3%'

-- Cirrhosis (liver is the primary organ for lactate clearance)

OR d.icd_code LIKE '571.5%'

-- Diabetic ketoacidosis (DKA, affects lactate metabolism)

OR d.icd_code LIKE '250.1%'

)) OR

(d.icd_version = 10 AND (

-- Epilepsy / seizures

d.icd_code LIKE 'G40%' OR d.icd_code LIKE 'G41%' OR d.icd_code LIKE 'R56%'

-- Alcohol-related conditions

OR d.icd_code LIKE 'F10%' OR d.icd_code LIKE 'K70%'

-- Cirrhosis

OR d.icd_code LIKE 'K74%'

-- Diabetic ketoacidosis (DKA)

OR d.icd_code LIKE 'E101%' OR d.icd_code LIKE 'E111%' OR d.icd_code LIKE 'E131%'

))

THEN 1 ELSE 0 END) AS is_lactate_metabolism,

-- Drug overdose (may cause distributive / cardiogenic shock)

MAX(CASE WHEN

(d.icd_version = 9 AND (

d.icd_code LIKE '960%' OR d.icd_code LIKE '961%' OR d.icd_code LIKE '962%'

OR d.icd_code LIKE '963%' OR d.icd_code LIKE '964%' OR d.icd_code LIKE '965%'

OR d.icd_code LIKE '966%' OR d.icd_code LIKE '967%' OR d.icd_code LIKE '968%'

OR d.icd_code LIKE '969%'

)) OR

(d.icd_version = 10 AND (

d.icd_code LIKE 'T36%' OR d.icd_code LIKE 'T37%' OR d.icd_code LIKE 'T38%'

OR d.icd_code LIKE 'T39%' OR d.icd_code LIKE 'T40%' OR d.icd_code LIKE 'T41%'

OR d.icd_code LIKE 'T42%' OR d.icd_code LIKE 'T43%' OR d.icd_code LIKE 'T44%'

OR d.icd_code LIKE 'T45%' OR d.icd_code LIKE 'T46%'

))

THEN 1 ELSE 0 END) AS is_drug_overdose,

-- DNR / comfort-care status

MAX(CASE WHEN

(d.icd_version = 9 AND (d.icd_code IN ('V667','V66.7','V4986','V49.86'))) OR

(d.icd_version = 10 AND (d.icd_code LIKE 'Z515%' OR d.icd_code LIKE 'Z66%'))

THEN 1 ELSE 0 END) AS is_dnr

FROM mimiciv_hosp.diagnoses_icd d

WHERE EXISTS (

SELECT 1 FROM t0_candidates t0

WHERE t0.hadm_id = d.hadm_id AND t0.hadm_id IS NOT NULL

)

GROUP BY d.hadm_id

),

-- Patients with non-septic shock diagnoses (applied exclusion)

excluded_diagnoses_non_dnr AS (

SELECT hadm_id

FROM combined_diag_scan

WHERE is_excluded_non_dnr = 1

),

-- Patients with conditions affecting lactate metabolism (scanned but NOT excluded)

excluded_lactate_metabolism AS (

SELECT hadm_id

FROM combined_diag_scan

WHERE is_lactate_metabolism = 1

),

-- Patients with drug overdose (applied exclusion)

excluded_drug_overdose AS (

SELECT hadm_id

FROM combined_diag_scan

WHERE is_drug_overdose = 1

),

-- Patients admitted from surgical service within 24 hours (applied exclusion)

excluded_recent_surgery AS (

SELECT DISTINCT t0.stay_id

FROM t0_candidates t0

INNER JOIN mimiciv_hosp.services srv

ON srv.hadm_id = t0.hadm_id

AND srv.curr_service IN ('CSURG', 'TSURG', 'VSURG', 'NSURG', 'ORTHO')

AND srv.transfertime BETWEEN t0.t0_time - INTERVAL '24 hours' AND t0.t0_time

),

-- Early comfort-focused care: DNR + death within 24 h + no vasopressor beyond 6 h

early_dnr_behavioral AS (

-- [Uniqueness] DISTINCT

SELECT DISTINCT t0.stay_id

FROM t0_candidates t0

INNER JOIN combined_diag_scan diag ON t0.hadm_id = diag.hadm_id

INNER JOIN mimiciv_hosp.patients pat ON t0.subject_id = pat.subject_id

WHERE

diag.is_dnr = 1

AND (pat.dod IS NOT NULL AND pat.dod >= t0.t0_time AND pat.dod < t0.t0_time + INTERVAL '24 hours')

AND NOT EXISTS (

SELECT 1 FROM mimiciv_icu.inputevents inp

WHERE inp.stay_id = t0.stay_id

AND inp.itemid IN (221906, 221289, 221662, 221749, 222315)

AND inp.rate > 0

AND (inp.statusdescription IS NULL

OR inp.statusdescription NOT IN ('Rewritten', 'Stopped', 'Paused', 'Flushed'))

AND inp.starttime <= t0.t0_time + INTERVAL '6 hours'

AND (

(inp.endtime IS NOT NULL AND inp.endtime >= t0.t0_time + INTERVAL '6 hours')

OR (inp.endtime IS NULL)

)

)

),

-- ============================================================================

-- Step 6: Hemoglobin data integration (multi-source)

-- ============================================================================

hb_all_sources_raw AS (

SELECT

t0.subject_id, t0.stay_id, t0.t0_time,

le.charttime AS hb_time,

le.valuenum AS hb_value,

'labevents' AS hb_source,

ABS(EXTRACT(EPOCH FROM (le.charttime - t0.t0_time))) AS time_diff_seconds,

CASE WHEN le.charttime <= t0.t0_time THEN 0 ELSE 1 END AS is_after_t0

FROM t0_candidates t0

INNER JOIN mimiciv_hosp.labevents le

ON t0.subject_id = le.subject_id

AND (le.hadm_id = t0.hadm_id OR le.hadm_id IS NULL)

AND le.charttime BETWEEN t0.t0_time - INTERVAL '48 hours' AND t0.t0_time + INTERVAL '6 hours'

AND le.itemid = 51222

AND le.valuenum BETWEEN 2 AND 20

UNION ALL

SELECT

t0.subject_id, t0.stay_id, t0.t0_time,

ce.charttime AS hb_time,

ce.valuenum AS hb_value,

'chartevents' AS hb_source,

ABS(EXTRACT(EPOCH FROM (ce.charttime - t0.t0_time))) AS time_diff_seconds,

CASE WHEN ce.charttime <= t0.t0_time THEN 0 ELSE 1 END AS is_after_t0

FROM t0_candidates t0

INNER JOIN mimiciv_icu.chartevents ce

ON t0.stay_id = ce.stay_id

AND ce.charttime BETWEEN t0.t0_time - INTERVAL '48 hours' AND t0.t0_time + INTERVAL '6 hours'

AND ce.itemid IN (220228, 227709)

AND ce.valuenum BETWEEN 2 AND 20

UNION ALL

SELECT

t0.subject_id, t0.stay_id, t0.t0_time,

le.charttime AS hb_time,

(le.valuenum / 3.0) AS hb_value,

'hct_estimated' AS hb_source,

ABS(EXTRACT(EPOCH FROM (le.charttime - t0.t0_time))) AS time_diff_seconds,

CASE WHEN le.charttime <= t0.t0_time THEN 0 ELSE 1 END AS is_after_t0

FROM t0_candidates t0

INNER JOIN mimiciv_hosp.labevents le

ON t0.subject_id = le.subject_id

AND (le.hadm_id = t0.hadm_id OR le.hadm_id IS NULL)

AND le.charttime BETWEEN t0.t0_time - INTERVAL '48 hours' AND t0.t0_time + INTERVAL '6 hours'

AND le.itemid = 51221

AND le.valuenum BETWEEN 6 AND 60

),

hb_best AS (

-- [Uniqueness] ROW_NUMBER + rn = 1

SELECT

subject_id, stay_id, t0_time,

hb_value, hb_source, hb_time,

ROUND(time_diff_seconds / 60.0, 1) AS time_diff_minutes,

is_after_t0

FROM (

SELECT *,

ROW_NUMBER() OVER (

PARTITION BY subject_id, stay_id, t0_time

ORDER BY

time_diff_seconds,

is_after_t0,

CASE hb_source

WHEN 'labevents' THEN 1

WHEN 'chartevents' THEN 2

WHEN 'hct_estimated' THEN 3

END

) AS rn

FROM hb_all_sources_raw

) ranked

WHERE rn = 1

),

-- Exclude patients who already had vasopressor infusion running before T0

early_vaso AS (

-- [Uniqueness] DISTINCT

SELECT DISTINCT t0.subject_id, t0.stay_id, t0.t0_time

FROM t0_candidates t0

INNER JOIN mimiciv_icu.inputevents inp

ON t0.stay_id = inp.stay_id

WHERE inp.itemid IN (221906, 221289, 221662, 221749, 222315)

AND inp.rate > 0

AND (inp.statusdescription IS NULL

OR inp.statusdescription NOT IN ('Rewritten', 'Stopped', 'Paused', 'Flushed'))

AND inp.starttime < t0.t0_time

AND inp.endtime >= t0.t0_time - INTERVAL '6 hours'

),

-- Apply exclusion criteria

t0_filtered AS (

SELECT t0.*

FROM t0_candidates t0

WHERE NOT EXISTS (SELECT 1 FROM excluded_diagnoses_non_dnr ex WHERE ex.hadm_id = t0.hadm_id)

-- Lactate-metabolism conditions were scanned but deliberately NOT excluded;

-- these patients (epilepsy, alcohol-related liver disease, cirrhosis, DKA)

-- remain in the cohort because LDR is intended for use in this population.

-- AND NOT EXISTS (SELECT 1 FROM excluded_lactate_metabolism lm WHERE lm.hadm_id = t0.hadm_id)

AND NOT EXISTS (SELECT 1 FROM excluded_drug_overdose od WHERE od.hadm_id = t0.hadm_id)

AND NOT EXISTS (SELECT 1 FROM excluded_recent_surgery rs WHERE rs.stay_id = t0.stay_id)

AND NOT EXISTS (SELECT 1 FROM early_dnr_behavioral dnr WHERE dnr.stay_id = t0.stay_id)

AND NOT EXISTS (SELECT 1 FROM early_vaso ev

WHERE ev.subject_id = t0.subject_id

AND ev.stay_id = t0.stay_id

AND ev.t0_time = t0.t0_time)

),

-- ============================================================================

-- Step 7: Invasive DBP extraction (itemid 220051, within 30 min before T0)

-- [Uniqueness] GROUP BY / DISTINCT ON

-- ============================================================================

dbp_at_t0 AS (

-- [Uniqueness] GROUP BY (subject_id, stay_id, t0_time)

SELECT

t0.subject_id, t0.stay_id, t0.t0_time,

AVG(ce.valuenum) AS dbp_avg,

MIN(ce.valuenum) AS dbp_min,

MIN(ce.charttime) AS dbp_measure_time,

COUNT(*) AS dbp_measurement_count

FROM t0_filtered t0

INNER JOIN mimiciv_icu.chartevents ce

ON t0.stay_id = ce.stay_id

AND ce.itemid = 220051

AND ce.charttime BETWEEN t0.t0_time - INTERVAL '30 minutes' AND t0.t0_time

AND ce.valuenum BETWEEN 20 AND 150

GROUP BY t0.subject_id, t0.stay_id, t0.t0_time

),

-- Closest DBP to T0

dbp_closest AS (

-- [Uniqueness] DISTINCT ON (subject_id, stay_id, t0_time)

SELECT DISTINCT ON (t0.subject_id, t0.stay_id, t0.t0_time)

t0.subject_id, t0.stay_id, t0.t0_time,

ce.valuenum AS dbp_at_t0,

ce.charttime AS dbp_closest_time

FROM t0_filtered t0

INNER JOIN mimiciv_icu.chartevents ce

ON t0.stay_id = ce.stay_id

AND ce.itemid = 220051

AND ce.charttime BETWEEN t0.t0_time - INTERVAL '30 minutes' AND t0.t0_time

AND ce.valuenum BETWEEN 20 AND 150

ORDER BY t0.subject_id, t0.stay_id, t0.t0_time,

ABS(EXTRACT(EPOCH FROM (ce.charttime - t0.t0_time))) ASC

),

-- ============================================================================

-- Step 8: Assemble baseline data with LDR calculation

-- [Uniqueness] All JOINs are on unique keys

-- ============================================================================

t0_with_basics AS (

SELECT

t0.subject_id,

t0.hadm_id,

t0.stay_id,

t0.sepsis_onset_time,

t0.trigger_lactate_time,

t0.trigger_lactate,

t0.t0_time,

-- DBP variables

dc.dbp_at_t0,

da.dbp_min,

ROUND(da.dbp_avg::NUMERIC, 1) AS dbp_avg,

da.dbp_measure_time,

da.dbp_measurement_count,

dc.dbp_closest_time,

-- LDR calculation (lactate / invasive DBP)

CASE

WHEN dc.dbp_at_t0 > 0 AND t0.trigger_lactate IS NOT NULL

THEN ROUND((t0.trigger_lactate / dc.dbp_at_t0)::NUMERIC, 4)

ELSE NULL

END AS ldr,

CASE

WHEN da.dbp_min > 0 AND t0.trigger_lactate IS NOT NULL

THEN ROUND((t0.trigger_lactate / da.dbp_min)::NUMERIC, 4)

ELSE NULL

END AS ldr_min_dbp,

CASE

WHEN da.dbp_avg > 0 AND t0.trigger_lactate IS NOT NULL

THEN ROUND((t0.trigger_lactate / da.dbp_avg)::NUMERIC, 4)

ELSE NULL

END AS ldr_avg_dbp,

-- Hemoglobin

hb.hb_value AS hemoglobin,

hb.hb_source AS hb_data_source,

hb.hb_time AS hb_measure_time,

hb.time_diff_minutes AS hb_time_diff_min,

CASE WHEN hb.is_after_t0 = 0 THEN 'before_T0' ELSE 'after_T0' END AS hb_timing,

-- Demographics and outcomes

pat.anchor_age + (EXTRACT(YEAR FROM t0.t0_time) - pat.anchor_year) AS age,

pat.gender,

pat.dod,

adm.hospital_expire_flag,

-- 28-day mortality from dod field

CASE

WHEN pat.dod IS NOT NULL AND pat.dod <= (t0.t0_time + INTERVAL '28 days')

THEN 1 ELSE 0

END AS mortality_28d,

-- Survival time in days

CASE

WHEN pat.dod IS NULL THEN NULL

WHEN DATE(pat.dod) = DATE(t0.t0_time) THEN 0.5

ELSE GREATEST(0.5, ROUND((EXTRACT(EPOCH FROM (pat.dod - t0.t0_time)) / 86400.0)::NUMERIC, 2))

END AS survival_days

FROM t0_filtered t0

INNER JOIN dbp_closest dc

ON t0.subject_id = dc.subject_id AND t0.stay_id = dc.stay_id AND t0.t0_time = dc.t0_time

LEFT JOIN dbp_at_t0 da

ON t0.subject_id = da.subject_id AND t0.stay_id = da.stay_id AND t0.t0_time = da.t0_time

LEFT JOIN hb_best hb

ON t0.subject_id = hb.subject_id AND t0.stay_id = hb.stay_id AND t0.t0_time = hb.t0_time

INNER JOIN mimiciv_hosp.patients pat ON t0.subject_id = pat.subject_id

INNER JOIN mimiciv_hosp.admissions adm ON t0.hadm_id = adm.hadm_id

WHERE t0.trigger_lactate IS NOT NULL

AND (pat.anchor_age + (EXTRACT(YEAR FROM t0.t0_time) - pat.anchor_year)) >= 18

),

-- Keep only the first ICU stay per patient

t0_first AS (

-- [Uniqueness] ROW_NUMBER PARTITION BY stay_id, filtered by rn_first = 1

SELECT *,

ROW_NUMBER() OVER (PARTITION BY stay_id ORDER BY t0_time ASC) AS rn_first

FROM t0_with_basics

),

-- ============================================================================

-- Step 9: Score component CTEs (all use GROUP BY for uniqueness)

-- ============================================================================

-- Arterial blood gas data

bg_combined AS (

-- [Uniqueness] GROUP BY (subject_id, stay_id, t0_time)

SELECT

t0.subject_id, t0.stay_id, t0.t0_time,

MIN(bg.pao2fio2ratio) AS pao2fio2ratio_min,

MIN(bg.ph) AS ph_min,

MAX(bg.ph) AS ph_max,

MIN(bg.po2) AS pao2_min,

MAX(bg.aado2) AS aado2_max,

MAX(CASE WHEN COALESCE(bg.fio2, 21) >= 50 THEN 1 ELSE 0 END) AS has_high_fio2

FROM t0_first t0

LEFT JOIN mimiciv_derived.bg bg

ON t0.hadm_id = bg.hadm_id

AND bg.charttime BETWEEN t0.t0_time - INTERVAL '24 HOUR' AND t0.t0_time + INTERVAL '2 HOUR'

AND bg.specimen = 'ART.'

WHERE t0.rn_first = 1

GROUP BY t0.subject_id, t0.stay_id, t0.t0_time

),

-- Complete blood count

cbc_combined AS (

-- [Uniqueness] GROUP BY (subject_id, stay_id, t0_time)

SELECT

t0.subject_id, t0.stay_id, t0.t0_time,

MIN(cbc.platelet) AS platelet_min,

MIN(cbc.hematocrit) AS hct_min,

MAX(cbc.hematocrit) AS hct_max,

MIN(cbc.wbc) AS wbc_min,

MAX(cbc.wbc) AS wbc_max

FROM t0_first t0

LEFT JOIN mimiciv_derived.complete_blood_count cbc

ON t0.hadm_id = cbc.hadm_id

AND cbc.charttime BETWEEN t0.t0_time - INTERVAL '24 HOUR' AND t0.t0_time + INTERVAL '2 HOUR'

WHERE t0.rn_first = 1

GROUP BY t0.subject_id, t0.stay_id, t0.t0_time

),

-- Chemistry panel

chem_combined AS (

-- [Uniqueness] GROUP BY (subject_id, stay_id, t0_time)

SELECT

t0.subject_id, t0.stay_id, t0.t0_time,

MAX(CASE WHEN chem.charttime >= t0.t0_time - INTERVAL '24 HOUR'

AND chem.charttime <= t0.t0_time + INTERVAL '2 HOUR'

THEN chem.creatinine END) AS creatinine_max,

MIN(CASE WHEN chem.charttime < t0.t0_time THEN chem.creatinine END) AS baseline_cr,

MIN(chem.bicarbonate) AS hco3_min,

MAX(chem.bicarbonate) AS hco3_max,

MIN(chem.sodium) AS sodium_min,

MAX(chem.sodium) AS sodium_max,

MIN(chem.potassium) AS potassium_min,

MAX(chem.potassium) AS potassium_max,

MIN(chem.creatinine) AS creatinine_min_apache,

MAX(chem.creatinine) AS creatinine_max_apache

FROM t0_first t0

LEFT JOIN mimiciv_derived.chemistry chem

ON t0.hadm_id = chem.hadm_id

AND chem.charttime BETWEEN t0.t0_time - INTERVAL '7 DAY' AND t0.t0_time + INTERVAL '2 HOUR'

WHERE t0.rn_first = 1

GROUP BY t0.subject_id, t0.stay_id, t0.t0_time

),

-- Bilirubin

bili_at_t0 AS (

-- [Uniqueness] GROUP BY (subject_id, stay_id, t0_time)

SELECT

t0.subject_id, t0.stay_id, t0.t0_time,

MAX(enz.bilirubin_total) AS bilirubin_max

FROM t0_first t0

LEFT JOIN mimiciv_derived.enzyme enz

ON t0.hadm_id = enz.hadm_id

AND enz.charttime BETWEEN t0.t0_time - INTERVAL '24 HOUR' AND t0.t0_time + INTERVAL '2 HOUR'

WHERE t0.rn_first = 1

GROUP BY t0.subject_id, t0.stay_id, t0.t0_time

),

-- Vital signs

vs_at_t0 AS (

-- [Uniqueness] GROUP BY (subject_id, stay_id, t0_time)

SELECT

t0.subject_id, t0.stay_id, t0.t0_time,

MIN(vs.mbp) AS meanbp_min,

MAX(vs.mbp) AS map_max,

MIN(vs.temperature) AS temp_min,

MAX(vs.temperature) AS temp_max,

MIN(vs.heart_rate) AS hr_min,

MAX(vs.heart_rate) AS hr_max,

MIN(vs.resp_rate) AS rr_min,

MAX(vs.resp_rate) AS rr_max

FROM t0_first t0

LEFT JOIN mimiciv_derived.vitalsign vs

ON t0.stay_id = vs.stay_id

AND vs.charttime BETWEEN t0.t0_time - INTERVAL '24 HOUR' AND t0.t0_time + INTERVAL '2 HOUR'

WHERE t0.rn_first = 1

GROUP BY t0.subject_id, t0.stay_id, t0.t0_time

),

-- Vasopressor rates at T0 and norepinephrine equivalent (NEE)

vaso_at_t0 AS (

-- [Uniqueness] GROUP BY (subject_id, stay_id, t0_time)

SELECT

t0.subject_id, t0.stay_id, t0.t0_time,

MAX(CASE WHEN mv.itemid = 221906 THEN mv.rate END) AS rate_norepinephrine,

MAX(CASE WHEN mv.itemid = 221289 THEN mv.rate END) AS rate_epinephrine,

MAX(CASE WHEN mv.itemid = 221662 THEN mv.rate END) AS rate_dopamine,

MAX(CASE WHEN mv.itemid = 221749 THEN mv.rate END) AS rate_phenylephrine,

MAX(CASE WHEN mv.itemid = 222315 THEN mv.rate END) AS rate_vasopressin,

COALESCE(MAX(CASE WHEN mv.itemid = 221906 THEN mv.rate END), 0)

+ COALESCE(MAX(CASE WHEN mv.itemid = 221289 THEN mv.rate END), 0)

+ COALESCE(MAX(CASE WHEN mv.itemid = 221662 THEN mv.rate END) / 100.0, 0)

+ COALESCE(MAX(CASE WHEN mv.itemid = 221749 THEN mv.rate END) / 10.0, 0)

+ COALESCE(MAX(CASE WHEN mv.itemid = 222315 THEN mv.rate END) * 2.5, 0)

AS nee,

COALESCE(MAX(CASE WHEN mv.itemid = 221906 THEN mv.rate END), 0)

+ COALESCE(MAX(CASE WHEN mv.itemid = 221289 THEN mv.rate END), 0)

+ COALESCE(MAX(CASE WHEN mv.itemid = 221749 THEN mv.rate END) / 10.0, 0)

+ COALESCE(MAX(CASE WHEN mv.itemid = 222315 THEN mv.rate END) * 2.5, 0)

AS nee_for_sofa

FROM t0_first t0

LEFT JOIN mimiciv_icu.inputevents mv

ON t0.stay_id = mv.stay_id

AND mv.starttime <= t0.t0_time + INTERVAL '5 MINUTE'

AND mv.endtime >= t0.t0_time - INTERVAL '5 MINUTE'

AND mv.itemid IN (221906, 221289, 221662, 221749, 222315)

AND mv.rate > 0

AND (mv.statusdescription IS NULL

OR mv.statusdescription NOT IN ('Rewritten', 'Stopped', 'Paused', 'Flushed'))

WHERE t0.rn_first = 1

GROUP BY t0.subject_id, t0.stay_id, t0.t0_time

),

-- Glasgow Coma Scale

gcs_at_t0 AS (

-- [Uniqueness] GROUP BY (subject_id, stay_id, t0_time)

SELECT

t0.subject_id, t0.stay_id, t0.t0_time,

MIN(gcs.gcs) AS gcs_min

FROM t0_first t0

LEFT JOIN mimiciv_derived.gcs gcs

ON t0.stay_id = gcs.stay_id

AND gcs.charttime BETWEEN t0.t0_time - INTERVAL '24 HOUR' AND t0.t0_time + INTERVAL '2 HOUR'

WHERE t0.rn_first = 1

GROUP BY t0.subject_id, t0.stay_id, t0.t0_time

),

-- Diagnosis flags for chronic disease

diag_status AS (

-- [Uniqueness] GROUP BY (subject_id, stay_id, t0_time)

SELECT

t0.subject_id, t0.stay_id, t0.t0_time,

MAX(CASE WHEN

(diag.icd_version = 9 AND diag.icd_code LIKE '585%') OR

(diag.icd_version = 10 AND diag.icd_code LIKE 'N18%')

THEN 1 ELSE 0 END) AS has_ckd,

MAX(CASE WHEN

(diag.icd_version = 9 AND (

diag.icd_code LIKE '571%' OR diag.icd_code LIKE '428%' OR

diag.icd_code LIKE '496%' OR diag.icd_code LIKE '585%' OR diag.icd_code LIKE '279%'

)) OR

(diag.icd_version = 10 AND (

diag.icd_code LIKE 'K70%' OR diag.icd_code LIKE 'K74%' OR

diag.icd_code LIKE 'I50%' OR diag.icd_code LIKE 'J44%' OR

diag.icd_code LIKE 'N18%' OR diag.icd_code LIKE 'D89%'

))

THEN 1 ELSE 0 END) AS has_chronic_disease

FROM t0_first t0

LEFT JOIN mimiciv_hosp.diagnoses_icd diag ON t0.hadm_id = diag.hadm_id

WHERE t0.rn_first = 1

GROUP BY t0.subject_id, t0.stay_id, t0.t0_time

),

-- Admission type and surgical status

-- [Fix 1] Removed adm.admission_type from GROUP BY; uses MAX aggregate instead

admission_status AS (

-- [Uniqueness] GROUP BY (subject_id, stay_id, t0_time)

SELECT

t0.subject_id, t0.stay_id, t0.t0_time,

MAX(CASE WHEN adm.admission_type = 'ELECTIVE' THEN 0 ELSE 1 END) AS is_emergency_admission,

MAX(CASE WHEN srv.curr_service IN (

'SURG', 'CSURG', 'TSURG', 'VSURG', 'ORTHO',

'TRAUM', 'NSURG', 'PSURG', 'GU', 'GYN'

) THEN 1 ELSE 0 END) AS has_surgery

FROM t0_first t0

INNER JOIN mimiciv_hosp.admissions adm ON t0.hadm_id = adm.hadm_id

LEFT JOIN mimiciv_hosp.services srv ON t0.hadm_id = srv.hadm_id

WHERE t0.rn_first = 1

GROUP BY t0.subject_id, t0.stay_id, t0.t0_time

),

-- ============================================================================

-- Step 10: Assemble all score components

-- ============================================================================

scorecomp AS (

SELECT

t0.subject_id, t0.hadm_id, t0.stay_id,

t0.sepsis_onset_time, t0.trigger_lactate_time, t0.trigger_lactate, t0.t0_time,

t0.dbp_at_t0, t0.dbp_min, t0.dbp_avg, t0.dbp_measure_time, t0.dbp_measurement_count,

t0.dbp_closest_time,

t0.ldr, t0.ldr_min_dbp, t0.ldr_avg_dbp,

t0.hemoglobin, t0.hb_data_source, t0.hb_measure_time, t0.hb_time_diff_min, t0.hb_timing,

t0.age, t0.gender, t0.dod, t0.hospital_expire_flag, t0.mortality_28d, t0.survival_days,

bg.pao2fio2ratio_min,

cbc.platelet_min,

bili.bilirubin_max,

vs.meanbp_min,

vaso.rate_norepinephrine, vaso.rate_epinephrine, vaso.rate_dopamine,

vaso.rate_phenylephrine, vaso.rate_vasopressin,

vaso.nee, vaso.nee_for_sofa,

gcs.gcs_min,

chem.creatinine_max, chem.baseline_cr,

vs.temp_min, vs.temp_max, vs.map_max, vs.hr_min, vs.hr_max, vs.rr_min, vs.rr_max,

bg.ph_min, bg.ph_max, bg.pao2_min, bg.aado2_max, bg.has_high_fio2,

chem.hco3_min, chem.hco3_max,

chem.sodium_min, chem.sodium_max, chem.potassium_min, chem.potassium_max,

chem.creatinine_min_apache, chem.creatinine_max_apache,

cbc.hct_min, cbc.hct_max, cbc.wbc_min, cbc.wbc_max,

-- Acute renal failure flag for APACHE II creatinine doubling

CASE

WHEN chem.baseline_cr IS NOT NULL THEN

CASE

WHEN chem.creatinine_max >= chem.baseline_cr * 1.5 THEN 1

WHEN chem.creatinine_max >= chem.baseline_cr + 0.3 THEN 1

ELSE 0

END

ELSE

CASE

WHEN chem.creatinine_max >= 1.5 AND COALESCE(ds.has_ckd, 0) = 0 THEN 1

ELSE 0

END

END AS has_arf,

ds.has_chronic_disease,

admstat.is_emergency_admission, admstat.has_surgery

FROM t0_first t0

LEFT JOIN bg_combined bg ON t0.subject_id = bg.subject_id AND t0.stay_id = bg.stay_id AND t0.t0_time = bg.t0_time

LEFT JOIN cbc_combined cbc ON t0.subject_id = cbc.subject_id AND t0.stay_id = cbc.stay_id AND t0.t0_time = cbc.t0_time

LEFT JOIN chem_combined chem ON t0.subject_id = chem.subject_id AND t0.stay_id = chem.stay_id AND t0.t0_time = chem.t0_time

LEFT JOIN bili_at_t0 bili ON t0.subject_id = bili.subject_id AND t0.stay_id = bili.stay_id AND t0.t0_time = bili.t0_time

LEFT JOIN vs_at_t0 vs ON t0.subject_id = vs.subject_id AND t0.stay_id = vs.stay_id AND t0.t0_time = vs.t0_time

LEFT JOIN vaso_at_t0 vaso ON t0.subject_id = vaso.subject_id AND t0.stay_id = vaso.stay_id AND t0.t0_time = vaso.t0_time

LEFT JOIN gcs_at_t0 gcs ON t0.subject_id = gcs.subject_id AND t0.stay_id = gcs.stay_id AND t0.t0_time = gcs.t0_time

LEFT JOIN diag_status ds ON t0.subject_id = ds.subject_id AND t0.stay_id = ds.stay_id AND t0.t0_time = ds.t0_time

LEFT JOIN admission_status admstat ON t0.subject_id = admstat.subject_id AND t0.stay_id = admstat.stay_id AND t0.t0_time = admstat.t0_time

WHERE t0.rn_first = 1

),

-- ============================================================================

-- Step 11: SOFA score calculation

-- ============================================================================

sofa_calc AS (

SELECT

scorecomp.*,

CASE

WHEN pao2fio2ratio_min < 100 THEN 4

WHEN pao2fio2ratio_min < 200 THEN 3

WHEN pao2fio2ratio_min < 300 THEN 2

WHEN pao2fio2ratio_min < 400 THEN 1

ELSE 0

END AS respiration,

CASE

WHEN platelet_min < 20 THEN 4

WHEN platelet_min < 50 THEN 3

WHEN platelet_min < 100 THEN 2

WHEN platelet_min < 150 THEN 1

ELSE 0

END AS coagulation,

CASE

WHEN bilirubin_max >= 12.0 THEN 4

WHEN bilirubin_max >= 6.0 THEN 3

WHEN bilirubin_max >= 2.0 THEN 2

WHEN bilirubin_max >= 1.2 THEN 1

ELSE 0

END AS liver,

CASE

WHEN COALESCE(rate_dopamine, 0) > 15 OR COALESCE(nee_for_sofa, 0) > 0.1 THEN 4

WHEN COALESCE(rate_dopamine, 0) > 5 OR (COALESCE(nee_for_sofa, 0) > 0 AND COALESCE(nee_for_sofa, 0) <= 0.1) THEN 3

WHEN COALESCE(rate_dopamine, 0) > 0 THEN 2

WHEN meanbp_min < 70 THEN 1

ELSE 0

END AS cardiovascular,

CASE

WHEN gcs_min = 15 OR gcs_min IS NULL THEN 0

WHEN gcs_min >= 13 THEN 1

WHEN gcs_min >= 10 THEN 2

WHEN gcs_min >= 6 THEN 3

ELSE 4

END AS cns,

CASE

WHEN creatinine_max >= 5.0 THEN 4

WHEN creatinine_max >= 3.5 THEN 3

WHEN creatinine_max >= 2.0 THEN 2

WHEN creatinine_max >= 1.2 THEN 1

ELSE 0

END AS renal

FROM scorecomp

),

-- ============================================================================

-- Step 12: APACHE II score calculation

-- ============================================================================

apache_calc AS (

SELECT

s.*,

GREATEST(

COALESCE(CASE

WHEN temp_max >= 41 THEN 4 WHEN temp_max >= 39 THEN 3 WHEN temp_max >= 38.5 THEN 1

WHEN temp_max >= 36 THEN 0 WHEN temp_max >= 34 THEN 1 WHEN temp_max >= 32 THEN 2

WHEN temp_max >= 30 THEN 3 WHEN temp_max < 30 THEN 4 ELSE NULL END, 0),

COALESCE(CASE

WHEN temp_min >= 41 THEN 4 WHEN temp_min >= 39 THEN 3 WHEN temp_min >= 38.5 THEN 1

WHEN temp_min >= 36 THEN 0 WHEN temp_min >= 34 THEN 1 WHEN temp_min >= 32 THEN 2

WHEN temp_min >= 30 THEN 3 WHEN temp_min < 30 THEN 4 ELSE NULL END, 0)

) AS temp_score,

GREATEST(

COALESCE(CASE

WHEN map_max >= 160 THEN 4 WHEN map_max >= 130 THEN 3 WHEN map_max >= 110 THEN 2

WHEN map_max >= 70 THEN 0 WHEN map_max >= 50 THEN 2 WHEN map_max < 50 THEN 4 ELSE NULL END, 0),

COALESCE(CASE

WHEN meanbp_min >= 160 THEN 4 WHEN meanbp_min >= 130 THEN 3 WHEN meanbp_min >= 110 THEN 2

WHEN meanbp_min >= 70 THEN 0 WHEN meanbp_min >= 50 THEN 2 WHEN meanbp_min < 50 THEN 4 ELSE NULL END, 0)

) AS map_score,

GREATEST(

COALESCE(CASE

WHEN hr_max >= 180 THEN 4 WHEN hr_max >= 140 THEN 3 WHEN hr_max >= 110 THEN 2

WHEN hr_max >= 70 THEN 0 WHEN hr_max >= 55 THEN 2 WHEN hr_max >= 40 THEN 3

WHEN hr_max < 40 THEN 4 ELSE NULL END, 0),

COALESCE(CASE

WHEN hr_min >= 180 THEN 4 WHEN hr_min >= 140 THEN 3 WHEN hr_min >= 110 THEN 2

WHEN hr_min >= 70 THEN 0 WHEN hr_min >= 55 THEN 2 WHEN hr_min >= 40 THEN 3

WHEN hr_min < 40 THEN 4 ELSE NULL END, 0)

) AS hr_score,

GREATEST(

COALESCE(CASE

WHEN rr_max >= 50 THEN 4 WHEN rr_max >= 35 THEN 3 WHEN rr_max >= 25 THEN 1

WHEN rr_max >= 12 THEN 0 WHEN rr_max >= 10 THEN 1 WHEN rr_max >= 6 THEN 2

WHEN rr_max < 6 THEN 4 ELSE NULL END, 0),

COALESCE(CASE

WHEN rr_min >= 50 THEN 4 WHEN rr_min >= 35 THEN 3 WHEN rr_min >= 25 THEN 1

WHEN rr_min >= 12 THEN 0 WHEN rr_min >= 10 THEN 1 WHEN rr_min >= 6 THEN 2

WHEN rr_min < 6 THEN 4 ELSE NULL END, 0)

) AS rr_score,

CASE

WHEN pao2fio2ratio_min < 100 THEN 4

WHEN pao2fio2ratio_min < 200 THEN 3

WHEN pao2fio2ratio_min < 300 THEN 2

WHEN pao2fio2ratio_min < 400 THEN 1

ELSE 0

END AS oxygenation_score,

GREATEST(

COALESCE(CASE

WHEN ph_max >= 7.7 THEN 4 WHEN ph_max >= 7.6 THEN 3 WHEN ph_max >= 7.5 THEN 1

WHEN ph_max >= 7.33 THEN 0 WHEN ph_max >= 7.25 THEN 2 WHEN ph_max >= 7.15 THEN 3

WHEN ph_max < 7.15 THEN 4 ELSE NULL END, 0),

COALESCE(CASE

WHEN ph_min >= 7.7 THEN 4 WHEN ph_min >= 7.6 THEN 3 WHEN ph_min >= 7.5 THEN 1

WHEN ph_min >= 7.33 THEN 0 WHEN ph_min >= 7.25 THEN 2 WHEN ph_min >= 7.15 THEN 3

WHEN ph_min < 7.15 THEN 4 ELSE NULL END, 0)

) AS acidbase_score,

GREATEST(

COALESCE(CASE

WHEN sodium_max >= 180 THEN 4 WHEN sodium_max >= 160 THEN 3 WHEN sodium_max >= 155 THEN 2

WHEN sodium_max >= 150 THEN 1 WHEN sodium_max >= 130 THEN 0 WHEN sodium_max >= 120 THEN 2

WHEN sodium_max >= 111 THEN 3 WHEN sodium_max < 111 THEN 4 ELSE NULL END, 0),

COALESCE(CASE

WHEN sodium_min >= 180 THEN 4 WHEN sodium_min >= 160 THEN 3 WHEN sodium_min >= 155 THEN 2

WHEN sodium_min >= 150 THEN 1 WHEN sodium_min >= 130 THEN 0 WHEN sodium_min >= 120 THEN 2

WHEN sodium_min >= 111 THEN 3 WHEN sodium_min < 111 THEN 4 ELSE NULL END, 0)

) AS sodium_score,

GREATEST(

COALESCE(CASE

WHEN potassium_max >= 7.0 THEN 4 WHEN potassium_max >= 6.0 THEN 3 WHEN potassium_max >= 5.5 THEN 1

WHEN potassium_max >= 3.5 THEN 0 WHEN potassium_max >= 3.0 THEN 1 WHEN potassium_max >= 2.5 THEN 2

WHEN potassium_max < 2.5 THEN 4 ELSE NULL END, 0),

COALESCE(CASE

WHEN potassium_min >= 7.0 THEN 4 WHEN potassium_min >= 6.0 THEN 3 WHEN potassium_min >= 5.5 THEN 1

WHEN potassium_min >= 3.5 THEN 0 WHEN potassium_min >= 3.0 THEN 1 WHEN potassium_min >= 2.5 THEN 2

WHEN potassium_min < 2.5 THEN 4 ELSE NULL END, 0)

) AS potassium_score,

CASE

WHEN creatinine_max_apache IS NULL THEN 0

ELSE

(CASE

WHEN creatinine_max_apache >= 3.5 THEN 4

WHEN creatinine_max_apache >= 2.0 THEN 3

WHEN creatinine_max_apache >= 1.5 THEN 2

WHEN creatinine_max_apache >= 0.6 THEN 0

WHEN creatinine_max_apache < 0.6 THEN 2

ELSE 0

END) * (CASE WHEN has_arf = 1 THEN 2 ELSE 1 END)

END AS creatinine_score,

GREATEST(

COALESCE(CASE

WHEN hct_max >= 60 THEN 4 WHEN hct_max >= 50 THEN 2 WHEN hct_max >= 46 THEN 1

WHEN hct_max >= 30 THEN 0 WHEN hct_max >= 20 THEN 2 WHEN hct_max < 20 THEN 4 ELSE NULL END, 0),

COALESCE(CASE

WHEN hct_min >= 60 THEN 4 WHEN hct_min >= 50 THEN 2 WHEN hct_min >= 46 THEN 1

WHEN hct_min >= 30 THEN 0 WHEN hct_min >= 20 THEN 2 WHEN hct_min < 20 THEN 4 ELSE NULL END, 0)

) AS hct_score,

GREATEST(

COALESCE(CASE

WHEN wbc_max >= 40 THEN 4 WHEN wbc_max >= 20 THEN 2 WHEN wbc_max >= 15 THEN 1

WHEN wbc_max >= 3 THEN 0 WHEN wbc_max >= 1 THEN 2 WHEN wbc_max < 1 THEN 4 ELSE NULL END, 0),

COALESCE(CASE

WHEN wbc_min >= 40 THEN 4 WHEN wbc_min >= 20 THEN 2 WHEN wbc_min >= 15 THEN 1

WHEN wbc_min >= 3 THEN 0 WHEN wbc_min >= 1 THEN 2 WHEN wbc_min < 1 THEN 4 ELSE NULL END, 0)

) AS wbc_score,

CASE WHEN gcs_min IS NULL THEN 0 ELSE 15 - gcs_min END AS gcs_score,

CASE

WHEN age IS NULL THEN 0

WHEN age >= 75 THEN 6 WHEN age >= 65 THEN 5 WHEN age >= 55 THEN 3 WHEN age >= 45 THEN 2

ELSE 0

END AS age_score,

CASE

WHEN has_surgery = 1 THEN

CASE WHEN has_chronic_disease = 1 AND is_emergency_admission = 1 THEN 5

WHEN has_chronic_disease = 1 THEN 2

ELSE 0 END

ELSE

CASE WHEN has_chronic_disease = 1 THEN 5

ELSE 0 END

END AS chronic_health_score

FROM sofa_calc s

),

-- ============================================================================

-- Step 13: Final score aggregation

-- ============================================================================

final_scores AS (

SELECT

a.*,

(COALESCE(respiration, 0) + COALESCE(coagulation, 0) + COALESCE(liver, 0) +

COALESCE(cardiovascular, 0) + COALESCE(cns, 0) + COALESCE(renal, 0)) AS sofa_score,

(COALESCE(temp_score, 0) + COALESCE(map_score, 0) + COALESCE(hr_score, 0) +

COALESCE(rr_score, 0) + COALESCE(oxygenation_score, 0) + COALESCE(acidbase_score, 0) +

COALESCE(sodium_score, 0) + COALESCE(potassium_score, 0) + COALESCE(creatinine_score, 0) +

COALESCE(hct_score, 0) + COALESCE(wbc_score, 0) + COALESCE(gcs_score, 0)) AS aps_score,

(COALESCE(temp_score, 0) + COALESCE(map_score, 0) + COALESCE(hr_score, 0) +

COALESCE(rr_score, 0) + COALESCE(oxygenation_score, 0) + COALESCE(acidbase_score, 0) +

COALESCE(sodium_score, 0) + COALESCE(potassium_score, 0) + COALESCE(creatinine_score, 0) +

COALESCE(hct_score, 0) + COALESCE(wbc_score, 0) + COALESCE(gcs_score, 0) +

COALESCE(age_score, 0) + COALESCE(chronic_health_score, 0)) AS apache2_score

FROM apache_calc a

),

-- [Fix 2] Deduplicate charlson table to ensure one row per hadm_id

charlson_dedup AS (

SELECT DISTINCT ON (hadm_id)

hadm_id,

charlson_comorbidity_index

FROM mimiciv_derived.charlson

ORDER BY hadm_id, charlson_comorbidity_index DESC

),

-- Aggregate all ICD diagnosis codes per admission

diagnosis_codes AS (

SELECT

d.hadm_id,

STRING_AGG(DISTINCT d.icd_code || ' (ICD' || d.icd_version || ')', '; ' ORDER BY d.icd_code || ' (ICD' || d.icd_version || ')') AS all_icd_codes

FROM mimiciv_hosp.diagnoses_icd d

WHERE EXISTS (

SELECT 1 FROM final_scores fs WHERE fs.hadm_id = d.hadm_id

)

GROUP BY d.hadm_id

)

-- ============================================================================

-- Final output

-- ============================================================================

SELECT

-- Identifiers

fs.subject_id, fs.hadm_id, stay_id,

sepsis_onset_time, trigger_lactate_time, trigger_lactate, t0_time,

-- DBP variables (invasive arterial, itemid 220051)

dbp_at_t0, dbp_min, dbp_avg, dbp_measure_time, dbp_measurement_count, dbp_closest_time,

-- Invasive SBP closest to T0 (scalar subquery, itemid 220050)

(SELECT ce.valuenum

FROM mimiciv_icu.chartevents ce

WHERE ce.stay_id = fs.stay_id

AND ce.itemid = 220050

AND ce.charttime BETWEEN fs.t0_time - INTERVAL '30 minutes' AND fs.t0_time

AND ce.valuenum BETWEEN 40 AND 300

ORDER BY ABS(EXTRACT(EPOCH FROM (ce.charttime - fs.t0_time)))

LIMIT 1

) AS sbp_at_t0,

-- Heart rate closest to T0 (scalar subquery)

(SELECT ce.valuenum

FROM mimiciv_icu.chartevents ce

WHERE ce.stay_id = fs.stay_id

AND ce.itemid = 220045

AND ce.charttime BETWEEN fs.t0_time - INTERVAL '30 minutes' AND fs.t0_time

AND ce.valuenum BETWEEN 20 AND 300

ORDER BY ABS(EXTRACT(EPOCH FROM (ce.charttime - fs.t0_time)))

LIMIT 1

) AS hr_at_t0,

-- LDR variants

ldr, ldr_min_dbp, ldr_avg_dbp,

-- Hemoglobin

hemoglobin, hb_data_source, hb_measure_time, hb_time_diff_min, hb_timing,

-- Demographics and outcomes

age, gender, dod, hospital_expire_flag, mortality_28d, survival_days,

-- SOFA components

pao2fio2ratio_min AS pao2fio2ratio_vent_min,

platelet_min, bilirubin_max, meanbp_min,

rate_norepinephrine, rate_epinephrine, rate_dopamine, rate_phenylephrine, rate_vasopressin,

nee, gcs_min, creatinine_max,

-- SOFA score and subscores

respiration, coagulation, liver, cardiovascular, cns, renal, sofa_score,

-- APACHE II raw data

temp_min, temp_max, meanbp_min AS map_min, map_max, hr_min, hr_max, rr_min, rr_max,

ph_min, ph_max, hco3_min, hco3_max, pao2_min, aado2_max,

sodium_min, sodium_max, potassium_min, potassium_max,

creatinine_max_apache, hct_min, hct_max, wbc_min, wbc_max,

has_arf, has_chronic_disease, has_surgery, is_emergency_admission,

-- APACHE II score and subscores

temp_score, map_score, hr_score, rr_score, oxygenation_score, acidbase_score,

sodium_score, potassium_score, creatinine_score, hct_score, wbc_score, gcs_score,

fs.age_score, chronic_health_score,

aps_score, apache2_score,

-- Charlson Comorbidity Index ([Fix 2] uses deduplicated table)

c.charlson_comorbidity_index AS cci_score,

-- All ICD diagnosis codes

dx.all_icd_codes

FROM final_scores fs

LEFT JOIN charlson_dedup c ON fs.hadm_id = c.hadm_id

LEFT JOIN diagnosis_codes dx ON fs.hadm_id = dx.hadm_id;

-- ============================================================================

-- Indexes on the output table

-- ============================================================================

CREATE INDEX IF NOT EXISTS idx_ssc_scores_subject ON mimiciv_derived.septic_shock_cohort_with_scores(subject_id);

CREATE INDEX IF NOT EXISTS idx_ssc_scores_stay ON mimiciv_derived.septic_shock_cohort_with_scores(stay_id);

CREATE INDEX IF NOT EXISTS idx_ssc_scores_hadm ON mimiciv_derived.septic_shock_cohort_with_scores(hadm_id);

CREATE INDEX IF NOT EXISTS idx_ssc_scores_mortality ON mimiciv_derived.septic_shock_cohort_with_scores(mortality_28d);

CREATE INDEX IF NOT EXISTS idx_ssc_scores_sofa ON mimiciv_derived.septic_shock_cohort_with_scores(sofa_score);

CREATE INDEX IF NOT EXISTS idx_ssc_scores_apache2 ON mimiciv_derived.septic_shock_cohort_with_scores(apache2_score);

CREATE INDEX IF NOT EXISTS idx_ssc_scores_ldr ON mimiciv_derived.septic_shock_cohort_with_scores(ldr);

-- ============================================================================

-- Table comment

-- ============================================================================

COMMENT ON TABLE mimiciv_derived.septic_shock_cohort_with_scores IS

'Septic shock cohort with SOFA and APACHE II scores, v3.9 (invasive arterial BP)

========================================

v3.9 fixes:

1. admission_status: removed admission_type from GROUP BY, uses MAX aggregate

2. charlson table: deduplicated with DISTINCT ON before JOIN

3. All CTEs annotated with uniqueness guarantees

New fields:

- sbp_at_t0: closest invasive SBP to T0 (scalar subquery)

- hr_at_t0: closest heart rate to T0 (scalar subquery)

Invasive arterial BP version:

- DBP: itemid 220051 (invasive diastolic blood pressure)

- SBP: itemid 220050 (invasive systolic blood pressure)

- Time window: 30 minutes before T0

Uniqueness guarantees:

- t0_candidates: ROW_NUMBER PARTITION BY (subject_id, stay_id)

- dbp_closest: DISTINCT ON (subject_id, stay_id, t0_time)

- All score CTEs: GROUP BY (subject_id, stay_id, t0_time)

- charlson_dedup: DISTINCT ON (hadm_id)

- sbp_at_t0, hr_at_t0: scalar subqueries with LIMIT 1';
