# Supplementary material for: Development and external validation of a parsimonious lactate-to-diastolic blood pressure ratio model for 28-day mortality risk stratification in septic shock: a retrospective two-cohort study
Source: Front Med (Lausanne). 2026 Jun 15;13:1827447. doi: 10.3389/fmed.2026.1827447 (PMC13311116; doi:10.3389/fmed.2026.1827447)
Supplement: Supplementary Material 1 — Additional methods, tables, and figures (including data extraction and variable definitions, additional head-to-head comparisons, threshold diagnostic performance, and sensitivity analyses). [file Table_1.docx]

# Supplementary material 1

This file contains Supplementary material 1 for submission to Frontiers.

# Contents of Supplementary material 1

| **ID** | **Title** | **Summary** |
| --- | --- | --- |
| S1 | Invasive vs noninvasive blood pressure | Comparison of LDR discrimination under two blood pressure measurement modalities and its intended scope |
| S2 | Dynamic changes in LDR | Predictive value of 6-h LDR clearance |
| S3 | Mechanistic exploration (Pcv-aCO₂) | Correlation between LDR and the Pcv-aCO₂ gap |
| S4 | Comparison of LDR with other metrics | Discrimination of LDR versus lactate, DSI, SOFA, and APACHE II |
| S5 | Missing-data analysis for key variables | Missingness and multiple-imputation methods in the two cohorts |
| S6 | Diagnostic performance of the cutoff LDR×100 ≥6.45 | Diagnostic performance of LDR×100=6.45 in the full cohort and the lactate gray-zone subgroup |
| S7 | Quick conversion table for high-risk LDR threshold | Quick table: high-risk DBP thresholds for common lactate values |
| S8 | Sensitivity analysis: lactate-T0 time window | Baseline characteristics and sensitivity analyses across lactate-T0 time windows (Tables S8-1, S8-2 and Figure S8-1) |
| S9 | Subgroup analysis: limited heart rate response | LDR vs DSI in the heart rate-limited subgroup (Tables S9-1, S9-2 and Figure S9-1) |
| S10 | Stratified analysis by hypertension history | LDR vs lactate stratified by hypertension history (Table S10) |
| S11 | Calibration assessment and recalibration | Calibration intercept and slope in the validation cohort and the linear recalibration formula |
| S12 | Exploratory multivariable comparison | Sanity check: AUC of LDR×100 alone vs LDR×100 + age + SOFA + NEE + CCI in both cohorts (Table S12) |
| S13 | Incremental reclassification (NRI and IDI) | Continuous NRI and IDI of LDR×100 vs single-variable lactate in both cohorts (Table S13) |

## S1 Performance of LDR using invasive versus noninvasive blood pressure measurements

Using the MIMIC-IV database, we compared the predictive performance of LDR between patients with invasive arterial blood pressure monitoring (n=962) and those with noninvasive blood pressure monitoring only (n=844; cuff DBP only at T₀). The noninvasive-only group comprised the subset of the 1,619 patients excluded from the primary validation cohort for lacking invasive DBP within 30 minutes before T₀ who nevertheless had a noninvasive cuff DBP recorded at T₀, and was analyzed separately to assess potential selection bias and generalizability. The coefficient of variation of DBP was substantially higher in the noninvasive-only group (31.9%) than in the invasive group (21.9%). LDR based on invasive blood pressure achieved an AUC of 0.714, which was higher than that in the noninvasive-only group (AUC 0.633; ΔAUC=0.081) (Tables S1-1 and S1-2).

These results suggest that LDR should be positioned as a baseline risk stratification tool in settings with invasive arterial monitoring. In clinical settings that rely on cuff blood pressure (e.g., emergency departments or general wards), LDR should not be considered equivalent to its performance in ICU invasive-monitoring environments. At present, we do not recommend directly extrapolating the LDR model developed under invasive monitoring to noninvasive blood pressure settings.

**Table S1-1. Baseline characteristics: invasive versus noninvasive blood pressure groups**

| **Variable** | **Invasive group (n=962)** | **Noninvasive group (n=844)** |
| --- | --- | --- |
| Age (years) | 65.8 ± 15.5 | 65.0 ± 15.9 |
| SOFA score | 8.0 ± 3.0 | 7.6 ± 3.1 |
| Lactate (mmol/L) | 4.37 ± 2.94 | 4.64 ± 3.15 |
| DBP (mmHg) | 55.3 ± 13.0 | 54.5 ± 17.4 |
| DBP coefficient of variation | 21.9% | 31.9% |
| LDR×100 | 8.46 ± 7.20 | 9.62 ± 7.91 |
| 28-day mortality | 26.0% | 48.6% |

**Table S1-2. Discrimination (AUC) of predictors in invasive versus noninvasive groups**

| **Metric** | **Invasive-group AUC (95% CI)** | **Noninvasive-group AUC (95% CI)** | **ΔAUC** |
| --- | --- | --- | --- |
| LDR | 0.714 (0.674-0.753) | 0.633 (0.604-0.662) | 0.081 |
| Lactate | 0.685 (0.644-0.727) | 0.639 (0.610-0.668) | 0.046 |
| DSI | 0.662 (0.621-0.704) | 0.547 (0.511-0.585) | 0.115 |
| SOFA | 0.687 (0.648-0.725) | 0.635 (0.601-0.670) | 0.052 |

*Note: In the noninvasive-only group, the AUC of LDR was significantly lower than in the invasive group. Mortality differed substantially between groups (48.6% vs 26.0%), suggesting selection bias.*

**Table S1-3. Chronic disease burden and functional status: invasive versus noninvasive monitoring groups**

| **Metric** | **Invasive group (n=962)** | **Noninvasive-only group (n=844)** | **Fold** |
| --- | --- | --- | --- |
| CCI | 4.8±2.7 | 5.6±2.9 | - |
| Dementia | 2.6% | 8.5% | 3.3× |
| From a care facility | 0.3% | 2.0% | 6.7× |
| End-stage renal disease (ESRD) | 1.3% | 4.4% | 3.4× |
| Cirrhosis | 1.2% | 3.5% | 2.9× |
| Metastatic cancer | 1.9% | 5.9% | 3.1× |
| DNR within 24 h after T0 | 1.3% | 3.8% | 2.9× |

*Note: CCI,* Charlson Comorbidity Index; ESRD, end-stage renal disease; DNR, do-not-resuscitate order. “From a care facility” includes nursing homes, rehabilitation centers, and skilled nursing facilities.

The higher mortality in the noninvasive-only monitoring group (48.6% vs 26.0%) was not driven by greater acute illness severity, but rather reflected implicit palliative selection based on chronic disease burden, functional status, and overall prognosis. As shown in Table S1-3, the noninvasive-only group had markedly higher proportions of indicators associated with poor long-term prognosis compared with the invasive group: 3.3-fold higher dementia prevalence, 6.7-fold higher proportion from a care facility, 3.4-fold higher ESRD, 2.9-fold higher cirrhosis, and 3.1-fold higher metastatic cancer. In addition, the noninvasive-only group more frequently transitioned to DNR status within 24 hours after T0.

These factors are not fully captured by conventional acute severity scores (e.g., SOFA or APACHE II) but influence clinicians’ decisions regarding monitoring intensity. Choosing noninvasive rather than invasive monitoring can partially reflect clinicians’ judgment of overall prognosis. Excluding the noninvasive-only group inevitably introduces selection bias because this group had higher mortality. We therefore restrict the LDR model's intended use to patients in whom invasive arterial monitoring is already indicated for hemodynamic management, rather than as a population-wide screening tool, and we transparently report the reduced LDR discrimination in the noninvasive-only subgroup (AUC 0.633) as a sensitivity analysis. Generalizability to settings without invasive arterial monitoring requires dedicated prospective evaluation.

## S2 Dynamic changes in LDR

We analyzed LDR changes among 257 patients in the development cohort with complete data at both T0 and T6. Six-hour LDR clearance was defined as: (LDR_T0 - LDR_T6) / LDR_T0 × 100%.

The absolute LDR values at either time point (T0 or T6) discriminated survivors from non-survivors (T0 AUC=0.692; T6 AUC=0.639), whereas 6-hour LDR clearance had no independent predictive value (AUC=0.501, near random). This suggests that DBP tends to become more homogeneous after vasopressor therapy, reducing the value of clearance as a dynamic monitoring marker. Accordingly, LDR is better positioned as a baseline risk stratification tool rather than a dynamic resuscitation monitoring metric (Table S2).

**Table S2. Dynamic changes in LDR (development cohort)**

| **Metric** | **Survivors (n=170)** | **Non-survivors (n=87)** | **P value** |
| --- | --- | --- | --- |
| T0 LDR×100 | 7.29 ± 3.77 | 12.34 ± 9.00 | <0.001 |
| T6 LDR×100 | 6.05 ± 3.75 | 10.33 ± 9.17 | <0.001 |
| 6-h LDR clearance (%) | 10.8 ± 50.7 | 12.6 ± 47.9 | 0.984 |
| AUC of 6-h LDR clearance | - | - | 0.501 |
| T0 lactate (mmol/L) | 3.95 ± 1.82 | 6.06 ± 4.38 | <0.001 |
| T6 lactate (mmol/L) | 3.67 ± 2.25 | 6.07 ± 5.13 | 0.001 |
| Lactate clearance (%) | 2.63 ± 55.85 | 0.44 ± 42.07 | 0.207 |
| T0 DBP (mmHg) | 57.2 ± 12.4 | 51.9 ± 14.9 | 0.001 |
| T6 DBP (mmHg) | 63.5 ± 12.8 | 61.1 ± 14.2 | 0.087 |

*Note: Clearance = (T0 value - T6 value) / T0 value × 100%. P values were calculated using the Mann-Whitney U test.*

## S3 Exploratory mechanistic analysis (Pcv-aCO₂ gap substudy)

In the development cohort, 51 patients had available Pcv-aCO₂ gap data. Pearson correlation showed a moderate positive association between LDR and the Pcv-aCO₂ gap (r=0.520, P<0.001). After adjusting for lactate (partial correlation), the association remained significant (r=0.412, P=0.003). These findings support the physiologic hypothesis that LDR is related to microcirculatory dysfunction (Table S3-1).

**Table S3-1. Correlation between LDR and the Pcv-aCO₂ gap (n=51)**

| **Analysis** | **Correlation coefficient (r)** | **95% CI** | **P value** |
| --- | --- | --- | --- |
| Pearson correlation (unadjusted) | 0.520 | 0.285-0.697 | <0.001 |
| Partial correlation (adjusted for lactate) | 0.412 | 0.153-0.618 | 0.003 |

*Note:* Pcv-aCO₂ represents the central venous-to-arterial CO₂ partial pressure difference. The partial correlation remained significant after adjustment for lactate, suggesting that the association between LDR and microcirculatory dysfunction is independent of tissue hypoxia as reflected by lactate.

We further performed joint risk stratification using LDR and Pcv-aCO₂, using cutoffs of LDR×100=6.45 and Pcv-aCO₂=6 mmHg to classify patients into four groups (Table S3-2):

**Table S3-2. Joint risk stratification using LDR and Pcv-aCO₂ (n=51)**

| **Group** | **n** | **Deaths** | **28-day mortality** |
| --- | --- | --- | --- |
| Both low (LDR×100 <6.45 and Pcv-aCO₂ <6) | 22 | 2 | 9.1% |
| Low LDR + high Pcv-aCO₂ | 8 | 2 | 25.0% |
| High LDR + low Pcv-aCO₂ | 6 | 2 | 33.3% |
| Both high (LDR×100 ≥6.45 and Pcv-aCO₂ ≥6) | 15 | 9 | 60.0% |

*Note: The cutoff of LDR×100=6.45 was derived from the optimal Youden index, and the cutoff of* Pcv-aCO₂=6 mmHg was based on published recommendations. Mortality in the “both high” group was 6.6 times that in the “both low” group.

## S4 Comparison of discrimination between LDR and other clinical metrics

To compare discrimination with commonly used clinical metrics, we compared ROC curves for LDR versus lactate, the diastolic shock index (DSI), the SOFA score, and the APACHE II score (Table S4).

**Table S4. AUC comparison for predicting 28-day mortality: LDR versus other metrics**

| **Metric** | **Development-cohort AUC (95% CI)** | **P vs LDR** | **Validation-cohort AUC (95% CI)** | **P vs LDR** |
| --- | --- | --- | --- | --- |
| LDR×100 | 0.726 (0.657-0.786) | - | 0.714 (0.674-0.753) | - |
| Lactate | 0.676 (0.604-0.747) | 0.006 | 0.685 (0.644-0.727) | 0.012 |
| SOFA | 0.734 (0.675-0.792) | 0.831 | 0.687 (0.648-0.725) | 0.270 |
| APACHE II | 0.717 (0.656-0.776) | 0.826 | 0.713 (0.677-0.749) | 0.973 |
| DSI | 0.651 (0.577-0.725) | 0.040 | 0.662 (0.621-0.704) | 0.043 |

*Note: P values are from paired DeLong tests. DSI, diastolic shock index (heart rate/DBP).*

## S5 Missing-data analysis for key variables

There were no missing data for the primary exposure (LDR×100) or the primary outcome (28-day mortality) in either cohort. In the validation cohort, heart rate was missing in 28 patients (2.9%), which affected DSI calculation (Tables S5-1 and S5-2).

**Table S5-1. Missingness of key variables in the two cohorts**

| **Variable** | **Development cohort (n=320)** | **Validation cohort (n=962)** |
| --- | --- | --- |
| LDR×100 (primary exposure) | 0 (0%) | 0 (0%) |
| Lactate | 0 (0%) | 0 (0%) |
| Diastolic blood pressure (DBP) | 0 (0%) | 0 (0%) |
| Heart rate (for DSI) | 0 (0%) | 28 (2.9%) |
| 28-day mortality (primary outcome) | 0 (0%) | 0 (0%) |

**Table S5-2. Missingness of other variables in the validation cohort**

| **Variable** | **Available (n)** | **Missing (n)** | **Missing (%)** |
| --- | --- | --- | --- |
| SOFA score | 962 | 0 | 0.00 |
| APACHE II score | 962 | 0 | 0.00 |
| Platelets | 939 | 23 | 2.39 |
| Creatinine | 950 | 12 | 1.25 |
| GCS | 902 | 60 | 6.24 |
| PaO₂/FiO₂ ratio | 866 | 96 | 9.98 |
| Bilirubin | 497 | 465 | 48.34 |
| Temperature | 823 | 139 | 14.45 |

*Note: The primary analyses (LDR×100, lactate, DBP, 28-day mortality) used complete cases because these variables had no missingness in either cohort. For auxiliary covariates used in sensitivity analyses (e.g., platelets, creatinine, GCS, PaO₂/FiO₂ ratio, bilirubin, temperature), multiple imputation by chained equations (MICE, m=20) was applied; primary results were unchanged.*

For covariates with missingness, multiple imputation by chained equations (MICE) was performed with m=20 imputed datasets and 20 iterations, using predictive mean matching for continuous variables and multinomial logistic regression for categorical variables. The imputation model included all analysis variables and auxiliary variables associated with missingness (lactate, DBP, age, SOFA, total bilirubin, creatinine, and—for the validation cohort—white-cell count). The primary univariate LDR×100 logistic model was re-estimated across imputed datasets and pooled by Rubin's rules to confirm equivalence with the complete-case analysis (Table S5-3). Because lactate, invasive DBP, LDR×100, and 28-day mortality had no missingness in either cohort, imputation was not required for the primary LDR model itself; MICE was used only for descriptive and auxiliary-covariate sensitivity analyses. Heart rate missingness in the validation cohort affected only the DSI calculation and did not impact the primary LDR analyses.

**Table S5-3. Univariate LDR×100 logistic regression: complete-case vs MICE with Rubin's rules pooling, in both cohorts.**

| **Metric** | **Validation CC (n=962)** | **Validation MICE (n=962)** | **Development CC (n=320)** | **Development MICE (n=320)** |
| --- | --- | --- | --- | --- |
| **β (LDR×100)** | 0.14277 | 0.14277 | 0.17200 | 0.17200 |
| **SE** | 0.01589 | 0.01589 | 0.02826 | 0.02826 |
| **OR (95% CI)** | 1.1535 (1.1181–1.1900) | 1.1535 (1.1181–1.1900) | 1.1877 (1.1237–1.2553) | 1.1877 (1.1237–1.2553) |
| **AUC (95% CI for CC; mean across imputations for MICE)** | 0.7136 (0.6710–0.7527) | 0.7136 | 0.7260 (0.6569–0.7857) | 0.7260 |
| **Brier score** | 0.1644 | 0.1644 | 0.1728 | 0.1728 |
| **FMI for β(LDR×100)** | — | 0.0000 | — | 0.0000 |
| **Δβ (MICE − CC)** | 0.00000 | 0.00000 | 0.00000 | 0.00000 |
| **ΔAUC (MICE − CC)** | 0.0000 | 0.0000 | 0.0000 | 0.0000 |

*Note. Coefficients, standard errors, odds ratios, AUC, and Brier scores were numerically identical (to 5 decimal places) between complete-case and MICE-pooled analyses in both cohorts. This is mathematically expected: because the primary model variables (LDR×100 and 28-day mortality) had zero missingness, the univariate logistic likelihood is identical across all imputed datasets, and the fraction of missing information (FMI) for the LDR×100 coefficient was 0 in both cohorts. The MICE procedure imputed only the auxiliary covariates, which do not enter the univariate primary model. CI = confidence interval; OR = odds ratio; AUC = area under the receiver-operating-characteristic curve; SE = standard error; FMI = fraction of missing information.*

## S6 Diagnostic performance of the cutoff LDR×100 ≥ 6.45

**Table S6. Diagnostic performance metrics for LDR×100=6.45 in the full cohort and in the lactate gray-zone subgroup.**

| **Cohort** | **Subgroup** | **n** | **Deaths** | **Prevalence** | **Sensitivity (95% CI)** | **Specificity (95% CI)** | **PPV (95% CI)** | **NPV (95% CI)** | **+LR (95% CI)** | **-LR (95% CI)** |
| --- | --- | --- | --- | --- | --- | --- | --- | --- | --- | --- |
| Development | Full cohort | 320 | 95 | 29.7% | 73.7% (64.2–82.5) | 62.2% (55.9–68.5) | 45.2% (37.7–52.9) | 84.8% (79.2–90.4) | 1.95 (1.60–2.42) | 0.42 (0.28–0.58) |
| Development | Gray-zone subgroup | 196 | 39 | 19.9% | 43.6% (27.3–60.0) | 87.9% (82.2–92.6) | 47.2% (30.3–63.2) | 86.2% (80.5–91.3) | 3.60 (2.02–6.52) | 0.64 (0.45–0.83) |
| Validation | Full cohort | 962 | 250 | 26.0% | 72.0% (66.3–77.5) | 59.4% (55.7–63.1) | 38.4% (34.0–42.8) | 85.8% (82.5–88.6) | 1.77 (1.57–1.99) | 0.47 (0.38–0.57) |
| Validation | Gray-zone subgroup | 592 | 103 | 17.4% | 35.0% (25.5–44.3) | 82.2% (78.6–85.5) | 29.3% (21.1–37.1) | 85.7% (82.5–88.8) | 1.96 (1.37–2.68) | 0.79 (0.67–0.91) |

*Note: The cutoff 6.45 was derived from the optimal Youden index in the full development cohort and should be regarded as exploratory pending prospective validation. 95% confidence intervals for sensitivity, specificity, PPV, NPV, +LR, and -LR were obtained by 2,000-iteration nonparametric bootstrap. PPV, positive predictive value; NPV, negative predictive value; +LR, positive likelihood ratio; -LR, negative likelihood ratio. The lactate gray-zone subgroup was defined as 2.0 ≤ lactate < 4.0 mmol/L.*

## S7 Quick conversion table for the high-risk LDR threshold

*Note: Table S7 provides a quick conversion table for high-risk DBP thresholds corresponding to common lactate values to facilitate bedside calculation.*

**Table S7. Quick conversion table for high-risk DBP thresholds**

| **Lactate (mmol/L)** | **High-risk DBP threshold (mmHg)** | **Clinical interpretation** |
| --- | --- | --- |
| 2.5 | ≤39 | DBP ≤39 mmHg indicates high risk |
| 3.0 | ≤47 | DBP ≤47 mmHg indicates high risk |
| 4.0 | ≤62 | DBP ≤62 mmHg indicates high risk |
| 5.0 | ≤78 | DBP ≤78 mmHg indicates high risk |
| 6.0 | ≤93 | DBP ≤93 mmHg indicates high risk |

*Note: High-risk DBP threshold = lactate×100÷6.45 ≈ lactate×15.5, corresponding to LDR×100 ≥6.45. This table is intended for invasive arterial pressure measurements and is not applicable to noninvasive settings.*

## S8 Sensitivity analysis: lactate-T0 time window

We performed a sensitivity analysis to assess whether the choice of pre-T0 lactate window affected LDR×100 discrimination. The validation cohort was stratified by the lactate-to-T0 interval (≤30, 31–60, 61–90, 91–120, and 121–180 minutes), and a sensitivity cohort restricted to lactate measured within 60 minutes before T0 (n=460) was compared with the full cohort (n=962, ≤180 minutes). Baseline characteristics across time-window strata are shown in Table S8-1; AUC comparisons between the full and sensitivity cohorts are shown in Table S8-2 and Figure S8-1.

**Table S8-1. Baseline characteristics stratified by the lactate-T0 time window (validation cohort)**

| **Time** | **n** | **Mort. %** | **Lactate (mmol/L)** | **DBP (mmHg)** | **LDR×100** | **SOFA** |
| --- | --- | --- | --- | --- | --- | --- |
| <=30min | 175 | 38.9 | 5.52 | 55.0 | 10.82 | 8.5 |
| 31-60min | 285 | 22.1 | 4.18 | 55.9 | 7.82 | 7.5 |
| 61-90min | 242 | 21.9 | 4.27 | 55.7 | 8.12 | 8.1 |
| 91-120min | 141 | 24.1 | 4.03 | 55.0 | 7.88 | 8.2 |
| 121-180min | 119 | 26.9 | 3.76 | 54.1 | 7.90 | 8.3 |


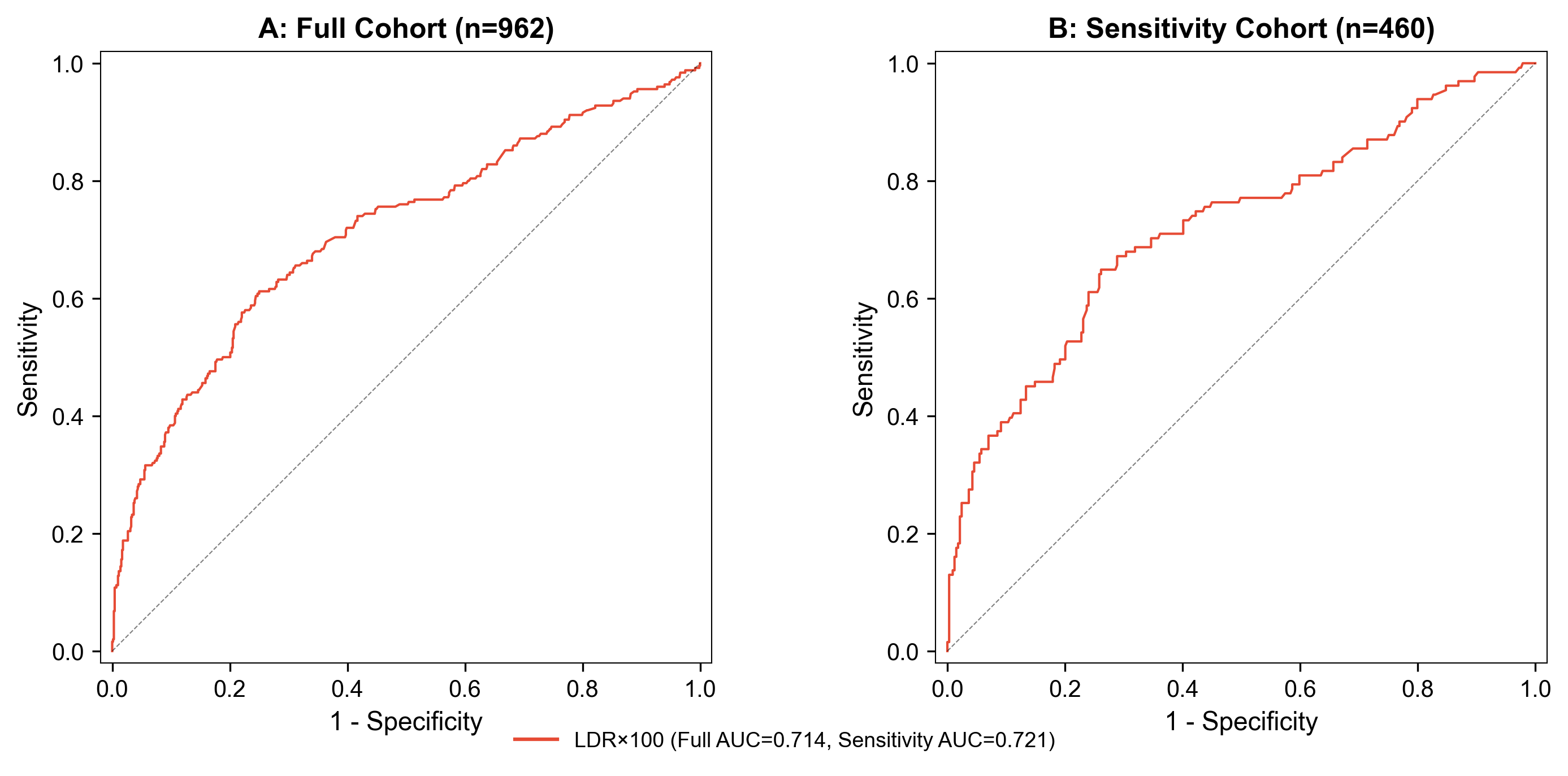
**Figure S8-1. ROC curves for the time-window sensitivity analysis (A: full cohort; B: sensitivity cohort)**

**Table S8-2. Sensitivity analysis by the lactate-T0 time window**

| **Cohort** | **n** | **Deaths** | **Mortality (%)** | **LDR AUC (95% CI)** |
| --- | --- | --- | --- | --- |
| Full cohort (≤180 min) | 962 | 250 | 26.0 | 0.714 (0.674-0.753) |
| Sensitivity cohort (≤60 min) | 460 | 131 | 28.5 | 0.721 (0.667-0.776) |

*Note: The AUC difference between the full cohort and the sensitivity cohort was 0.007, suggesting limited impact of time-window differences on the main conclusions.*

## S9 Subgroup analysis: limited heart rate response

**Key finding: the discrimination advantage of LDR is amplified in the heart rate-limited subgroup.**

**Table S9-1. Limited heart rate response subgroup: discrimination comparison of LDR versus DSI**

| **Cohort** | **Subgroup** | **n** | **Deaths** | **LDR AUC** | **DSI AUC** | **ΔAUC** | **95% CI** | **P value** |
| --- | --- | --- | --- | --- | --- | --- | --- | --- |
| Development | HR<100 | 170 | 36 | 0.674 | 0.448 | +0.226 | +0.104~+0.349 | <0.001 |
|  | HR>=100 | 150 | 59 | 0.716 | 0.696 | +0.020 | -0.078~+0.118 | 0.694 |
| Validation | HR<100 | 625 | 116 | 0.756 | 0.601 | +0.155 | +0.085~+0.224 | <0.001 |
|  | HR>=100 | 309 | 126 | 0.648 | 0.575 | +0.073 | +0.001~+0.145 | 0.046 |

*Note: ΔAUC = AUC(LDR) - AUC(DSI). 95% CIs and P values are based on the DeLong test. Interaction effect (ΔAUC in the heart rate-limited group minus ΔAUC in the normal heart rate group): +0.206 in the development cohort and +0.082 in the validation cohort.*

Sensitivity analysis (HR <90 beats/min)

**Table S9-2. Sensitivity analysis using a stricter definition of limited heart rate response**

| **Cohort** | **Subgroup** | **n** | **LDR AUC** | **DSI AUC** | **ΔAUC** |
| --- | --- | --- | --- | --- | --- |
| Development | HR <90 (severely limited) | 86 | 0.609 | 0.484 | +0.125 |
|  | HR>=90 | 234 | 0.794 | 0.783 | +0.011 |
| Validation | HR <90 (severely limited) | 485 | 0.761 | 0.587 | +0.174 |
|  | HR>=90 | 449 | 0.672 | 0.612 | +0.060 |


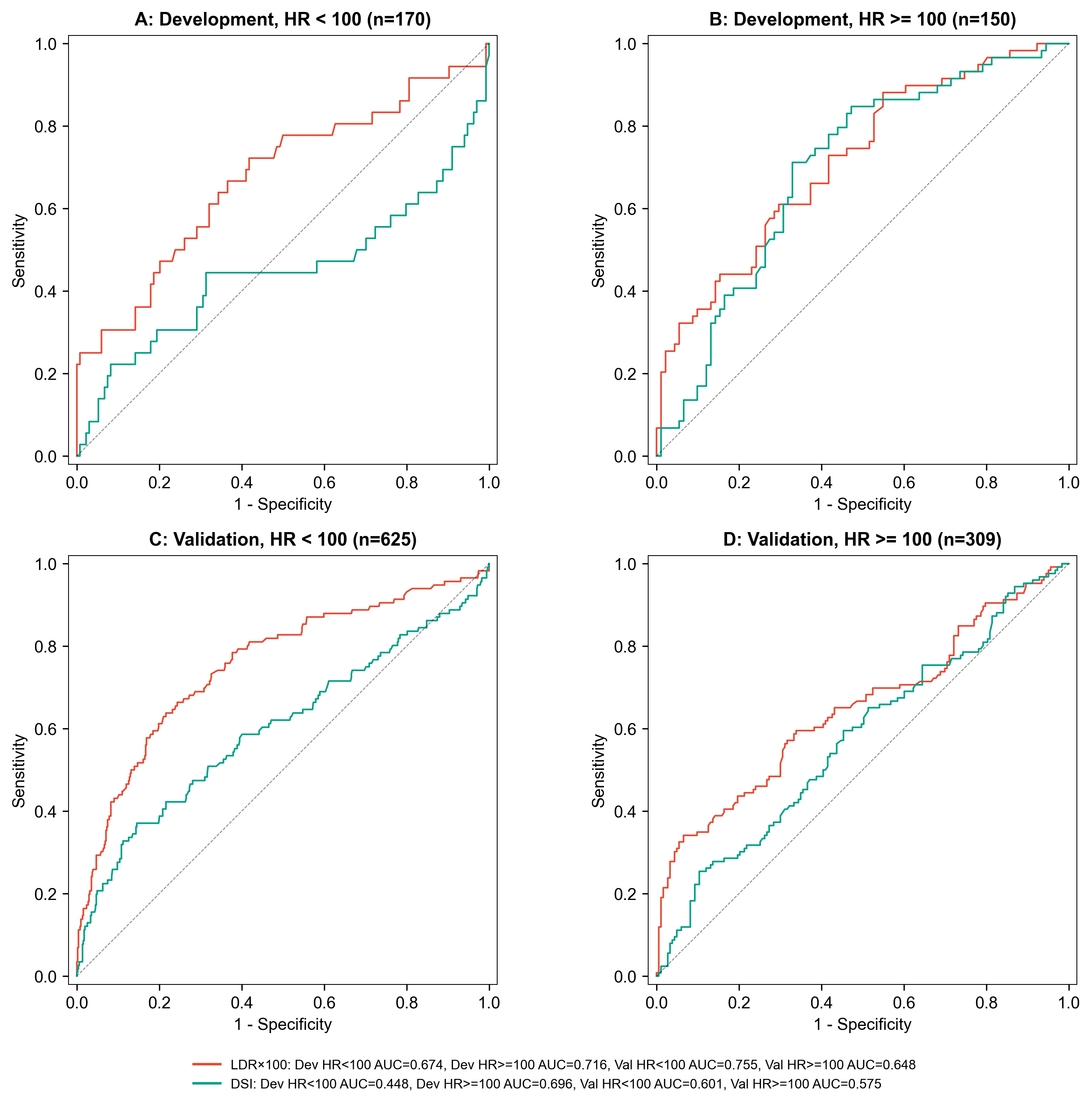


**Figure S9-1. ROC curve comparisons of LDR versus DSI stratified by heart rate**

## S10 Stratified analysis by hypertension history

To evaluate potential confounding by chronic vascular stiffening, we performed stratified analyses by hypertension history. In the development cohort, hypertension history was obtained from medical records; in the validation cohort, hypertension was defined using ICD-9 codes 401-405 or ICD-10 codes I10-I15. Results suggested that among patients without hypertension (relatively preserved vascular compliance), the predictive effect of LDR remained robust (Table S10).

**Table S10. Stratified analysis by hypertension history**

| **Cohort** | **Subgroup** | **n** | **Events** | **Mortality** | **LDR×100 AUC (95% CI)** | **Lactate AUC (95% CI)** | **ΔAUC** |
| --- | --- | --- | --- | --- | --- | --- | --- |
| Development | Overall | 320 | 95 | 29.7% | 0.726 (0.657-0.786) | 0.676 (0.606-0.747) | +0.050 |
|  | Hypertension | 193 | 52 | 26.9% | 0.701 (0.607-0.789) | 0.623 (0.521-0.724) | +0.079 |
|  | No hypertension | 127 | 43 | 33.9% | 0.755 (0.658-0.844) | 0.733 (0.634-0.830) | +0.021 |
| Validation | Overall | 962 | 250 | 26.0% | 0.714 (0.674-0.753) | 0.685 (0.644-0.727) | +0.029 |
|  | Hypertension | 608 | 150 | 24.7% | 0.707 (0.656-0.758) | 0.688 (0.635-0.741) | +0.019 |
|  | No hypertension | 354 | 100 | 28.2% | 0.722 (0.659-0.786) | 0.678 (0.611-0.746) | +0.044 |

*Note: In the development cohort, hypertension history was obtained from medical records. In the validation cohort, hypertension was defined using ICD-9 codes 401-405 or ICD-10 codes I10-I15. 95% CIs were estimated using bootstrap resampling (2,000 iterations). ΔAUC = AUC(LDR×100) -* AUC(lactate). Interaction tests: development cohort P=0.638 and validation cohort P=1.000 (logistic regression interaction term hypertension×LDR×100).

## S11 Calibration assessment and recalibration in the external validation cohort

In the external validation cohort, we assessed calibration using the original model linear predictor (LP = −2.365 + 0.172 × LDR×100) by fitting a logistic regression model logit(p) = a + b × LP to estimate the calibration intercept (a) and slope (b). If calibration drift is present, linear recalibration can be applied: LP__recal = a + b × LP, and the recalibrated probability is p_recal = 1 / (1 + exp(−LP_recal)_). In this study, the calibration intercept and slope in external validation were a = −0.335 and b = 0.830 (see Results in the main text).

A calibration slope of 0.830 indicates moderate overestimation at high predicted probabilities and underestimation at low probabilities — i.e., the model is overconfident in its extreme predictions. The original model therefore systematically miscalibrates absolute risk in the external validation setting, and should be used for rank-order risk stratification only. Local recalibration using the formula above is required before LDR×100 is used to estimate absolute 28-day mortality risk for clinical decision-making in new populations. Where local data are unavailable, LDR×100 may still be used for ordinal risk stratification (low / intermediate / high), since rank ordering is preserved even when absolute probabilities drift.

## S12 Exploratory multivariable comparison

As a sanity check addressing the possibility of underfitting from a single-predictor specification, we compared the parsimonious LDR×100 model against an exploratory multivariable extension that added age, SOFA score, norepinephrine equivalent dose (NEE) at T0, and the Charlson Comorbidity Index. Discrimination was assessed by AUC and compared using paired DeLong tests. The multivariable extension yielded statistically significant incremental discrimination in both cohorts (development: AUC 0.726 → 0.782, ΔAUC = +0.056, DeLong P = 0.028; validation: AUC 0.714 → 0.747, ΔAUC = +0.034, DeLong P = 0.039; Table S12). The absolute gain was modest, however, and required variables that are less suitable for immediate bedside triage (integrated severity scores and a comorbidity index that are typically not available at the moment of risk stratification). We therefore retained LDR×100 as a rapid risk-stratification trigger rather than a comprehensive prognostic model, while transparently reporting the multivariable comparison here.

**Table S12. Multivariable comparison: LDR×100 alone vs LDR×100 + age + SOFA + NEE + CCI**

| **Cohort** | **Model** | **AUC (95% CI)** | **ΔAUC vs LDR×100 alone** | **DeLong P** |
| --- | --- | --- | --- | --- |
| Development (n=320, 95 events) | LDR×100 alone | 0.726 (0.657–0.786) | — | — |
|  | LDR×100 + age + SOFA + NEE + CCI | 0.782 (0.722–0.838) | +0.056 | 0.028 |
| Validation (n=962, 250 events) | LDR×100 alone | 0.714 (0.674–0.753) | — | — |
|  | LDR×100 + age + SOFA + NEE + CCI | 0.747 (0.708–0.785) | +0.034 | 0.039 |

*Note: The multivariable extension was performed as an exploratory sensitivity analysis to assess whether the parsimonious single-predictor specification resulted in substantial loss of discrimination, and is not the primary model. AUCs were estimated by logistic regression in the development cohort and applied to the validation cohort. 95% CIs were obtained by 2,000 bootstrap replicates. ΔAUC was tested by paired DeLong test. The multivariable model improved discrimination in both cohorts at statistical significance (DeLong P = 0.028 and P = 0.039), but the absolute gain was modest and required integrated severity scores (SOFA, APACHE II) and a comorbidity index (CCI) that are typically not available at T0 for immediate bedside triage. LDR×100 was therefore retained as the primary model for use as a rapid risk-stratification trigger rather than a comprehensive prognostic model.*

## S13 Incremental reclassification (NRI and IDI)

To quantify the incremental value of LDR×100 over single-variable lactate, we computed the continuous net reclassification improvement (NRI) and the integrated discrimination improvement (IDI) per Pencina 2008. Both indices compare predicted probabilities from a single-variable logistic model on lactate (baseline) with those from a single-variable logistic model on LDR×100 (new), fit in the development cohort and applied to the validation cohort. 95% CIs were obtained by 2,000 bootstrap replicates.

Compared with lactate alone, LDR×100 achieved improved risk reclassification in both cohorts. In the development cohort, continuous NRI was 0.508 (95% CI 0.067 to 0.774) and IDI was 0.029 (95% CI -0.012 to 0.072). In the validation cohort, continuous NRI was 0.332 (95% CI 0.127 to 0.552) and IDI was 0.016 (95% CI 0.006 to 0.027). NRI reached statistical significance in both cohorts (P < 0.05); IDI was significant in the validation cohort but did not reach significance in the development cohort. This pattern — a significant ΔAUC in the development cohort (+0.050, P=0.006) but a non-significant IDI in the same cohort — is not contradictory: ΔAUC and IDI are distinct quantities (ΔAUC summarises rank-order discrimination over all decision thresholds, whereas IDI averages absolute differences in predicted probabilities between events and non-events), and IDI is known to have substantially higher sampling variance than ΔAUC in smaller cohorts. The wider confidence interval for IDI in the development cohort (n=320, 95 events) is consistent with the smaller event count and does not undermine the discrimination findings; both metrics point in the same direction, and both are significant in the larger validation cohort.

**Table S13. Reclassification metrics for LDR×100 vs single-variable lactate**

| **Cohort** | **Metric** | **Estimate (95% CI)** | **P value** |
| --- | --- | --- | --- |
| **Development (n=320, 95 events)** | Continuous NRI | 0.508 (0.067 to 0.774) | <0.05 |
|  | IDI | 0.029 (-0.012 to 0.072) | n.s. |
| **Validation (n=962, 250 events)** | Continuous NRI | 0.332 (0.127 to 0.552) | <0.05 |
|  | IDI | 0.016 (0.006 to 0.027) | <0.05 |

*Note: Baseline = single-variable logistic model on lactate; New = single-variable logistic model on LDR×100. Models were fit in the development cohort and applied to the validation cohort. Continuous NRI and IDI computed per Pencina et al. (2008); 95% CIs obtained by 2,000 bootstrap replicates. NRI, net reclassification improvement; IDI, integrated discrimination improvement.*
