# Supplementary material for: Development and external validation of a parsimonious lactate-to-diastolic blood pressure ratio model for 28-day mortality risk stratification in septic shock: a retrospective two-cohort study
Source: Front Med (Lausanne). 2026 Jun 15;13:1827447. doi: 10.3389/fmed.2026.1827447 (PMC13311116; doi:10.3389/fmed.2026.1827447)
Supplement: Supplementary Material 2 — TRIPOD checklist (completed). [file Table_2.docx]

**TRIPOD Checklist**

**Study Type:** Development and External Validation (Type 3)

**Manuscript:** Development and External Validation of a Parsimonious Lactate-to-Diastolic Blood Pressure Ratio (LDR) Model for 28-Day Mortality Risk Stratification in Septic Shock

| Section / Item No. | Checklist Item | Reported on Page/Section |
| --- | --- | --- |
| Title and abstract |  |  |
| Title — 1 | Identify the study as developing and/or validating a multivariable prediction model, the target population, and the outcome to be predicted. | Title page (Title) |
| Abstract — 2 | Provide a summary of objectives, study design, setting, participants, sample size, predictors, outcome, statistical analysis, results, and conclusions. | Submitted separately via the journal submission system |
| Introduction |  |  |
| Background and objectives — 3a | Explain the medical context (including whether diagnostic or prognostic) and rationale for developing or validating the multivariable prediction model, including references to existing models. | Introduction |
| 3b | Specify the objectives, including whether the study describes the development or validation of the model or both. | Introduction - study objectives |
| Methods |  |  |
| Source of data — 4a | Describe the study design or source of data (e.g., randomized trial, cohort, or registry data), separately for the development and validation data sets, if applicable. | Methods - Study design and ethics; Data sources and study population |
| 4b | Specify the key study dates, including start of accrual; end of accrual; and, if applicable, end of follow-up. | Methods - Data sources and study population |
| Participants — 5a | Specify key elements of the study setting (e.g., primary care, secondary care, general population) including number and location of centres. | Methods - Data sources and study population |
| 5b | Describe eligibility criteria for participants. | Methods - Data sources and study population |
| 5c | Give details of treatments received, if relevant. | Methods - Performance assessment (comparator indicators) |
| Outcome — 6a | Clearly define the outcome that is predicted by the prediction model, including how and when assessed. | Methods - Outcome definition |
| 6b | Report any actions to blind assessment of the outcome to be predicted. | Methods - Outcome definition (blinding not applicable; objective records) |
| Predictors — 7a | Clearly define all predictors used in developing or validating the multivariable prediction model, including how and when they were measured. | Methods - Predictor definition |
| 7b | Report any actions to blind assessment of predictors for the outcome and other predictors. | Methods - Predictor definition (blinding not applicable; objective records) |
| Sample size — 8 | Explain how the study size was arrived at. | Methods - Sample size |
| Missing data — 9 | Describe how missing data were handled (e.g., complete-case analysis, single imputation, multiple imputation) with details of any imputation method. | Methods - Missing data and statistical software; Supplementary material 1 (missing data/sensitivity analyses) |
| Statistical analysis methods — 10a | Describe how predictors were handled in the analyses. | Methods - Model development, external validation, and performance assessment |
| 10b | Specify type of model, all model-building procedures (including any predictor selection), and method for internal validation. | Methods - Validation approach (external validation performed; no separate internal validation) |
| 10c | For validation, describe how the predictions were calculated. | Methods - Model development, external validation, and performance assessment |
| 10d | Specify all measures used to assess model performance and, if relevant, to compare multiple models. | Methods - Performance assessment (AUC, calibration, decision curve analysis); Statistical analysis methods |
| 10e | Describe any model updating (e.g., recalibration) arising from the validation, if done. | Methods - Calibration (including linear recalibration); Supplementary material 1 (recalibration details) |
| Risk groups — 11 | Provide details on how risk groups were created, if done. | Methods - Four-quadrant stratification / risk grouping |
| Results |  |  |
| Participants — 13a | Describe the flow of participants through the study, including the number of participants with and without the outcome and, if applicable, a summary of the follow-up time. A diagram may be helpful. | Results - Participants and baseline characteristics; Figure 1 |
| 13b | Describe the characteristics of the participants (basic demographics, clinical features, available predictors), including the number of participants with missing data for predictors and outcome. | Results - Baseline characteristics; Table 1 |
| 13c | For validation, show a comparison with the development data of the distribution of important variables (demographics, predictors and outcome). | Results: Baseline characteristics; Table 1 |
| Model development — 14a | Specify the number of participants and outcome events in each analysis. | Results - Model performance; Table 2; Figures 2-4 |
| 14b | If done, report the unadjusted association between each candidate predictor and outcome. | Results - Model coefficients / OR; Table 2 |
| Model specification — 15a | Present the full prediction model to allow predictions for individuals (i.e., all regression coefficients, and model intercept or baseline survival at a given time point). | Results - Model specification (equation) |
| 15b | Explain how to the use the prediction model. | Discussion - Clinical application and suggested threshold; Supplementary Table S7 |
| Model performance — 16 | Report performance measures (with CIs) for the prediction model. | Results (Table 2; Figures 2-4; Table 3) |
| Model updating — 17 | If done, report the results from any model updating (i.e., model specification, model performance). | Results - Model specification and calibration (intercept/slope and linear recalibration); Figure 3 |
| Discussion |  |  |
| Limitations — 18 | Discuss any limitations of the study (such as nonrepresentative sample, few events per predictor, missing data). | Discussion - Limitations |
| Interpretation — 19a | For validation, discuss the results with reference to performance in the development data, and any other validation data. | Discussion - Main findings and interpretation |
| 19b | Give an overall interpretation of the results, considering objectives, limitations, results from similar studies, and other relevant evidence. | Discussion - Comparison with existing evidence / recalibration recommendations |
| Implications — 20 | Discuss the potential clinical use of the model and implications for future research. | Discussion - Clinical implications and future research; Conclusions |
| Other information |  |  |
| Supplementary information — 21 | Provide information about the availability of supplementary resources, such as study protocol, Web calculator, and data sets. | Data availability statement; Supplementary material |
| Funding — 22 | Give the source of funding and the role of the funders for the present study. | Declarations - Funding |
